# Nest characteristics determine nest microclimate and affect breeding output in an Antarctic seabird, the Wilson’s storm-petrel

**DOI:** 10.1371/journal.pone.0217708

**Published:** 2019-06-13

**Authors:** Rosanne J. Michielsen, Anne N. M. A. Ausems, Dariusz Jakubas, Michał Pętlicki, Joanna Plenzler, Judy Shamoun-Baranes, Katarzyna Wojczulanis-Jakubas

**Affiliations:** 1 Institute for Biodiversity and Ecosystem Dynamics, University of Amsterdam, Amsterdam, The Netherlands; 2 Department of Vertebrate Ecology and Zoology, University of Gdańsk, Gdańsk, Poland; 3 Centro de Estudios Científicos, Valdivia, Chile; 4 Institute of Biochemistry and Biophysics, Polish Academy of Sciences, Warsaw, Poland; Wagner College, UNITED STATES

## Abstract

The importance of nest characteristics for birds breeding in the extreme climate conditions of polar regions, has been greatly understudied. Nest parameters, like nest orientation, exposure and insulation, could strongly influence microclimate and protection against precipitation of the nest, thereby affecting breeding success. A burrow nesting seabird, the Wilson’s storm-petrel (*Oceanites oceanicus*) is an excellent model species to investigate the importance of nest characteristics, as it is the smallest endotherm breeding in the Antarctic. Here, we investigated the effects of nest parameters such as internal nest dimensions, nest micro-topography and thermal properties of the nest burrow and the influence of weather conditions on breeding output, measured as hatching success, chick survival, and chick growth. We collected data during the austral summers of 2017 and 2018, on King George Island, maritime Antarctica. Our results showed that the thermal microclimate of the nest burrow was significantly improved by a small entrance size, a low nest height, and insulation and tended to be enhanced by a low wind exposition index and an eastern nest site orientation. In addition, an eastern nest site orientation significantly reduced the chance of snow blocking. However, the relationships between nest characteristics and breeding output were complex and might be affected by other parameters like food availability and parental quality. The relation between chick growth and nest air temperature remained especially indistinct. Nevertheless, our results indicate that nest characteristics that enhance the thermal microclimate and reduce the risk of snow blocking favoured both hatching success and chick survival. Due to climate change in the Antarctic, snowfall is expected to increase in the future, which will likely enhance the importance of nest characteristics that determine snow blocking. Additionally, despite global warming, thermally favourable nest burrows will likely still be advantageous in the highly variable and challenging Antarctic climate.

## Introduction

Avian breeding success is determined by many variables such as parental effort, food availability, parasites, predation and nest characteristics [1–8]. Of those, the importance of nest characteristics has been understudied, especially for species that breed in harsh climates such as in the Antarctic zone. Nevertheless, previous research indicates that nest parameters like nest orientation, exposure and insulation play an important role in providing a thermally favourable microclimate and protection against precipitation (e.g. rain or snow), enhancing overall breeding success [6,9–16].

A suboptimal nest microclimate can be caused by various factors, such as extreme nest temperatures and exposure to wind or precipitation [17–21]. Suboptimal nest temperatures during incubation have been shown to have a negative impact on embryonic development [22,23]. In addition, increased energetic demands of incubating parents due to a low nest temperature may lead to reduced parental attentiveness and therewith increased periodic cooling of the clutch or the constant maintenance of clutch temperature below the developmental optimum, which is detrimental for the embryonic development [24–29]. Nest temperature may also affect chick growth and survival. For instance, low nest temperature has been shown to hamper the immune response of common yellowthroat (*Geothlypis trichas*) chicks [30]. Also, suboptimal thermal nest conditions caused by a high nest temperature have been shown to retard chick growth in blue tits (*Cyanistes caeruleus*) and eastern kingbirds (*Tyrannus tyrannus*) [31,32]. This highlights the importance of the thermal microclimate of the nest on breeding success.

Thermal microclimate of the nest is particularly important for endothermic vertebrates in regions with harsh climatic conditions such as low temperatures, strong winds and snow cover in alpine, Arctic and Antarctic regions. For example, chicks of little auks (*Alle alle*) breeding in the High-Arctic, grew faster in nest chambers with a higher mean air temperature [33]. In harsh polar habitats breeding is usually challenging and animals breeding in such circumstances exhibit various coping mechanims (reviewed by Martin and Wiebe [34]). Adults and chicks of avian species breeding in polar regions have been shown to exhibit high metabolic rates or energy requirements compared to species breeding in temperate environments [35–38]. Similarly, metabolic rates of Adélie penguin (*Pygoscelis adeliae*) chicks and blue-eyed shag (*Phalacrocorax atriceps*) chicks have been found to correlate negatively with ambient temperature [39]. In addition to low temperatures, snowfall during the breeding season has been reported as a major cause of egg and chick mortality and reduced body condition of the incubating parents or the chick for several species breeding in alpine, Arctic or Antarctic environments [2,34,40–44].

Under harsh conditions of extreme environments, the selection of a sheltered nest site and construction of insulating nest lining can greatly improve the microclimate in the nest, increasing nest temperature, reducing heat loss and the effects of snowfall [45–50]. Indeed, several Arctic breeding shorebird species have been found to select thermally favourable nest sites, and especially smaller shorebird species invested in the construction of nest lining [49]. For example, in the pectoral sandpiper (*Calidris melanotos*) breeding in the Arctic, thicker nest lining was associated with reduced heat loss of the clutch [46]. In common eiders (*Somateria mollissima*) breeding on the Arctic tundra, sheltered nests in combination with higher ambient temperature reduced the incubation effort (in terms of body mass loss of the incubating parent) [20].

A burrow nesting seabird, the Wilson’s storm-petrel (*Oceanites oceanicus*), is an excellent model species to investigate the importance of nest characteristics in extreme environments. First of all, it is the smallest endotherm breeding in the Antarctic, and due to the unfavourable volume-surface ratio of such a small body, both parents and chicks are thought to experience extreme energetic demands to maintain a constant body temperature [35,51,52]. In addition, breeding pairs have a maximum of one breeding attempt with a single egg per year, which increases their vulnerability to unfavourable conditions during the breeding season [53]. Moreover, food sources are unpredictable and usually require the parents to perform long foraging trips, during which the egg may be neglected or the chick is not fed for up to four days [51,54–56]. Hence, internal nest dimensions, nest micro-topography and nest insulation creating a thermally favourable nest burrow, are likely to greatly enhance the breeding success by reducing the costs of thermoregulation of Wilson’s storm-petrel parents and chicks, and to reduce the amount of egg cooling when the parents are absent. Furthermore, the nest burrows of the Wilson’s storm-petrel are prone to snow blocking (i.e. the nest entrance is completely blocked by snow), which can lead to breeding failure due starvation of the chick [43,53,57–59]. Therefore, nest site micro-topography that reduces the chance of snow blocking is likely to increase breeding success.

Here, we investigated the effect of nest parameters including internal nest dimensions, nest site micro-topography, (i.e. shelter provided by surrounding structures), and thermal properties of the nest burrow as well as with weather conditions on hatching success, chick survival, and chick growth rate in the Wilson’s storm-petrel. We first examined whether the thermal microclimate of the nest depends on the shelter provided by the nest burrow features (expressed by nest dimensions, entrance orientation and thermal insulation) and by the nest site micro-topography (expressed by exposure to the wind, terrain ruggedness and orientation of the nest site) in combination with weather conditions. Subsequently, we examined whether susceptibility of the nest to snow blocking depends on its micro-topography, i.e. nest orientation and nest exposure (expressed by size of the nest entrance, wind exposure and terrain ruggedness of the nest site) in combination with wind speed and wind direction, and the amount of snowfall. We expected that hatching success and chick growth would be affected by the nest parameters mainly through their influence on nest temperature. Since higher air temperature may slow down the cooling of the egg during the absence of the parent, and reduce thermoregulation costs of the incubating parent and developing chick, we hypothesized that higher nest air temperature would increase both hatching success and chick growth rate. We also expected that chick survival would be affected by nest parameters through a varying susceptibility to snow blocking, rather than through the nest air temperature. Based on that prediction, we further hypothesized that a higher risk of snow blocking of the nest would result in higher chick mortality.

## Materials and methods

### Study site

**Fig 1:**
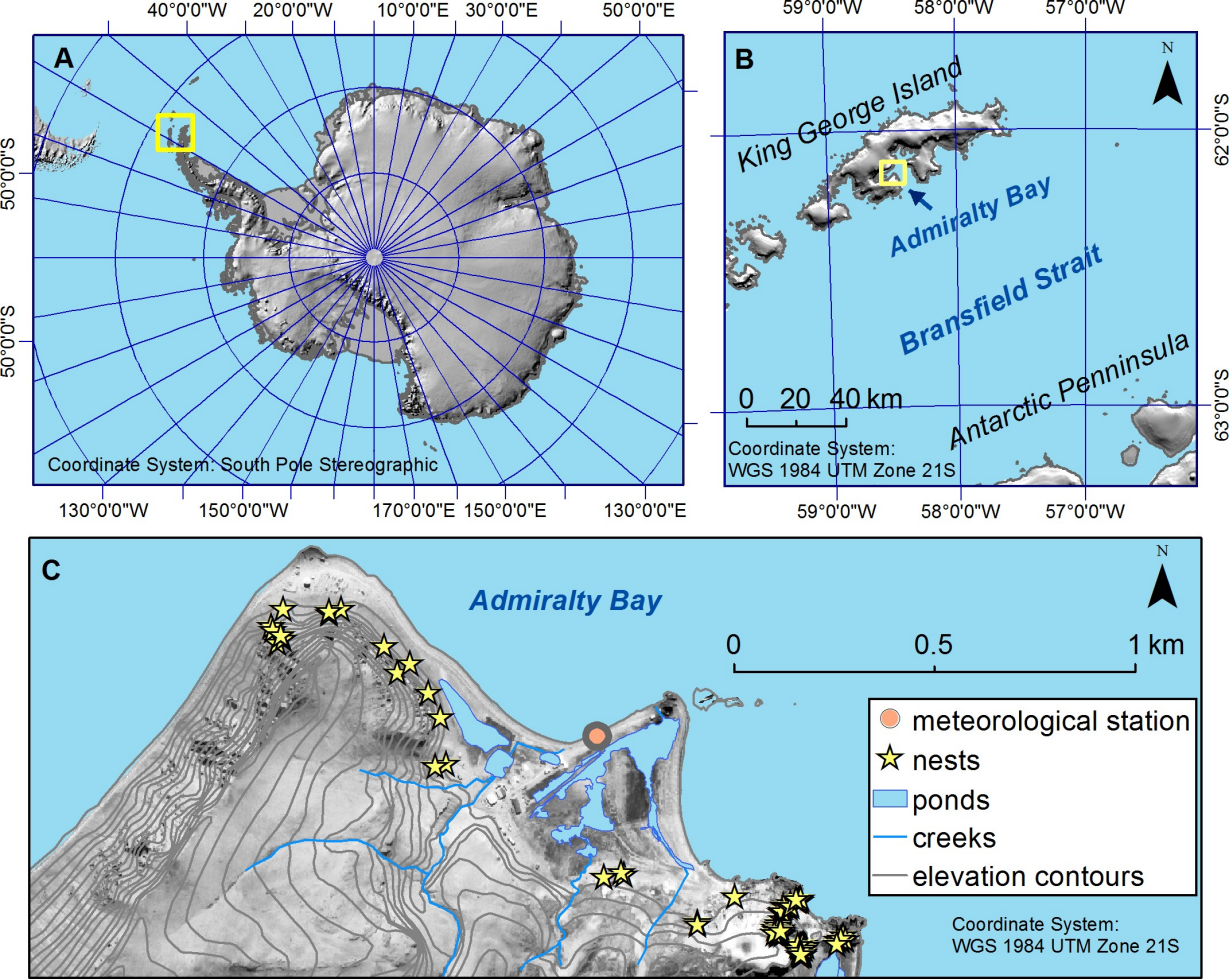
Location and an overview of the study area. Location in respect to the Antarctic continent (A; yellow rectangle) and King George Island (B; yellow rectangle), and an overview of the study area with the position of the 118 nests (C, yellow stars), and the meteorological station (C, orange circle).

We conducted our study in the Admiralty Bay area on King George Island (KGI), South Shetland Islands, maritime Antarctica (62°02′S 58°21′W, Fig 1) from mid-incubation (January) to the end of the chick rearing period (mid-April) of the Wilson’s storm-petrel, during the austral summers of 2017 and 2018. Although a major part of KGI is covered by glaciers [60], some coastal areas of the island are glacier free and accommodate numerous pinnipeds and seabirds. The weather conditions on KGI vary greatly between years, but in general are characterised by relatively low air temperatures (Table 1), high wind speeds (Table 1), and occasional snowfall during the austral summer [61]. We conducted the study in the area recognized as one of the main breeding aggregations of Wilson’s storm-petrels in the Admiralty Bay area [57,62].

**Table 1:** Description of weather conditions during the study period in 2017 and 2018: 27 January 2017–30 March 2017 (N_days_ = 60) and 15 January 2018–5 April 2018 (N_days_ = 80).

Parameter	Breeding season 2017 27 Jan– 28 Mar	Breeding season 2018 15 Jan– 5 Apr
Mean (min–max range) of air temperature (°C)	2.1 (-6.6–9.1)	2.3 (-7.5–9.6)
Proportion of days with precipitation	39%	83%
Proportion of days with snow cover	5.0%	24%
Predominant wind direction	North and west	Southwest and northwest
Mean wind speed (m∙s^-1^)	4.6	5.4
Proportion of days with wind speed > 5 m∙s^-1^	37.3%	57.3%

### Study species

The Wilson’s storm-petrel is a long-lived, small pelagic seabird that breeds in crevices in rock falls and among boulder scree along the Antarctic and sub-Antarctic coastline. Both parents share the incubation (~ 40 days) and chick rearing, (~ 60 days) [53,57]. Chicks are usually fed 1.2 times per 24 h [53] and are capable of thermoregulation in five days from hatching [52,63]. In periods of bad weather in combination with low food provisioning, nestlings are able to enter a state of hypothermia, to minimize metabolic activity and energy requirements [44,52,64].

To investigate the breeding success and chick growth, we determined the breeding status (i.e. the presence or absence of an egg or a chick) and the current state of the chick (i.e. dead or alive). In addition, we measured wing length (measured with a calliper, accurate to 0.1 mm), and chick body mass (measured with an electronic scale, OHAUS, USA, accurate to 0.1 g) during nest controls performed every 3 days (weather permitting) (details on further calculations on chick growth are provided in S2 File). In case of snow blocking the nest entrance (completely or partly), we did not extract the chick nor remove any snow. This was to avoid affecting the breeding success, for example due to accidently getting the chick wet, or damage the insulating properties of the snow cover. Instead, we postponed the chick measurements until the nest opening was naturally free of snow. We performed the study under the permission of the Polish National SCAR (to enter Special Antarctic Protected area no 128, and interfere of Antarctic fauna, no 6/2017 and 8/2017). Following European Union regulations EU regarding animal welfare and guidelines published by Animal Behaviour, the study did not require the agreement of the Animal Ethics Committee due to a minimal level of disturbance [65,66].

### Weather conditions

To examine the effect of weather conditions on the thermal microclimate in the nests, we measured air temperature, in °C, with a temperature probe (Vaisala, Finland, HMP155, accuracy: ± 0.2°C, range: -40 to 20°C, 2.0 m above ground level), wind speed, in m ∙ s^-1^, with an anemometer (Vector Instruments, UK, A100R, accuracy: ± 0.1 m ∙ s^-1^ at 0–10 m ∙ s^-1^, 1% between 10–55 m ∙ s^-1^ and 2% above 55 m ∙ s^-1^, 2.0 m above ground level) and wind direction, with an ultrasonic wind sensor (Gill Instruments, UK, WindSonic, accuracy: ± c2° at 12 m ∙ s^-1^, 2.5 m above ground level) and converted to the cosines of the radians: from north = 1 to south = -1 (hereafter northern wind direction,) or the sines of the radians: from east = 1 to west = -1 (hereafter eastern wind direction). We recorded weather conditions every 10 minutes, using an automated meteorological station (station and CR3000 data logger manufactured by Campbell Scientific, USA), situated in a flat, waterlogged area 100 m from the sea coast, and 1200 m from the furthest nest (Fig 1). We recorded the presence of precipitation (1 = higher or equal to 0.1 mm, 0 = lower than 0.1 mm, using a Hellman manual standard rain gauge) and snow cover (1 = any snow cover, continuous or discontinuous, 0 = no snow cover) measured several meters from the automatic weather station, once a day at 9:00 AM local time. Table 1 provides a description of the weather conditions during the study period.

### Nest site micro-topography

**Fig 2:**
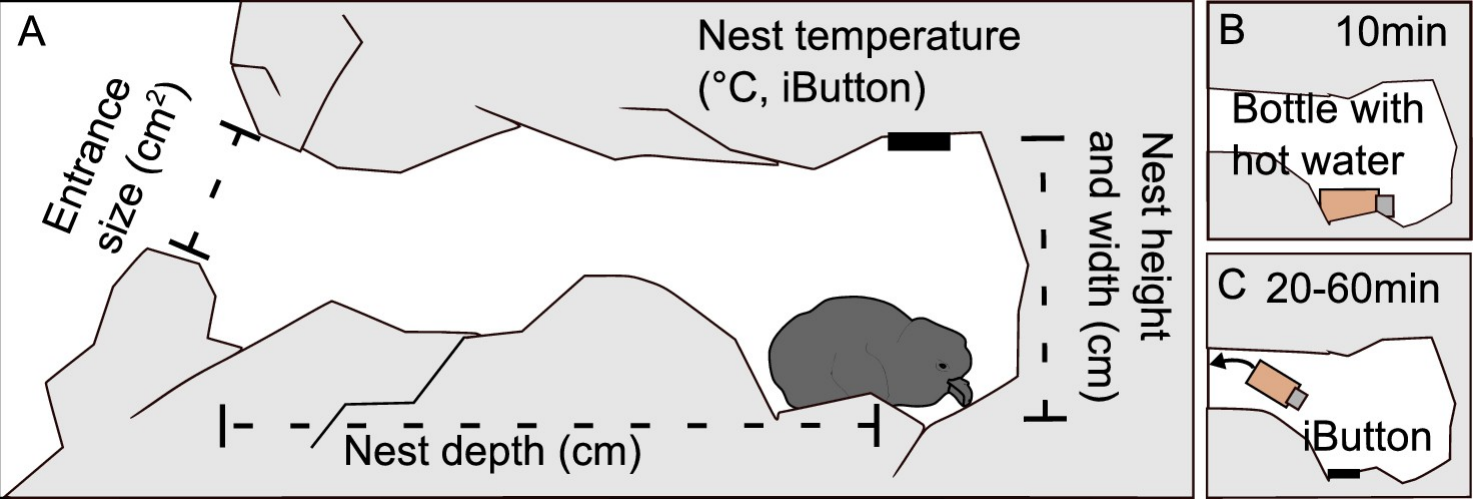
Overview of the study design. Measurements of the nest chamber (A) and the two phases of the cooling experiment: warming up the nest floor for 10 min with a bottle filled with hot water (B) and removing the bottle and placing a temperature logger (iButton) to record the floor temperature for a duration of 20–60 min (C) (sensitivity analysis of different measurement durations in S1 File).

We recorded coordinates of all the nests found in the two study seasons using a handheld GPS receiver (GPSMAP 64S, Garmin, USA, accuracy ± 3 m). For all the nests we recorded the snow blocking status (i.e. 1 = nest entrance was closed off by snow completely, 0 = nest entrance was open) during each nest check. To examine the shelter effect of the nest related to the nest size and its orientation, we measured three internal dimensions of nest chamber (in cm): depth (distance on the floor, from nest entrance to the centre of the nest floor), width (measured from the left centre point of the nest wall to the most distant opposite point of the chamber, crossing the nest cup), and height (measured from the centre of the nest floor to the opposite point of the nest ceiling, or the point where the temperature logger was attached) (Fig 2A). We also measured the size of the nest entrance (total area in cm^2^), based on photo analysis in ImageJ [67]: we took photos of the nest entrance with a ruler placed in the same plane of the entrance, and ensured the camera was positioned perpendicular to this plane. Finally, we measured the entrance orientation with a conventional pocket transit compass (0–360, Brunton, USA, accuracy: ± 1°, added 10° to measurement, to account for magnetic declination), then converted degrees to cosines of the radians [68] and classified as northern entrance orientation (from north = 1 to south = -1) or the sines of the radians and classified as the eastern entrance orientation (from east = 1 to west = -1) [68].

To quantify the shelter provided by the surroundings, we calculated the terrain ruggedness index (TRI, the amount of elevation change m^-1^) [69], the slope (meter elevation m^-1^), the nest site orientation, converted to the cosines of the radians, classified as the northern nest site orientation (from north = 1 to south = -1), and sines of the radians, classified as the eastern nest site orientation (from east = 1 to west = -1) and the wind exposition index (WEI, the level of wind exposure, <1 = shielded/concealed from wind, >1 = exposed to wind) [70]. To calculate these parameters, we used a digital elevation model based on an along-track, tri-stereo set of Pléiades 1A panchromatic images collected on 25 December 2012 (Centre National d’Etudes Spatiales, Paris, France) and a set of six ground control points. The sampling resolution was 0.5 m (see [71] for more details on the model). We set a search radius for the slope, aspect and TRI for 5 cells (2.5 m) corresponding to ~20 m^2^ circular footprint area. We calculated the WEI with a wind fetch length of 1 km. Some nest locations were snow covered during the satellite image acquisitions (~10% of all nest locations), the TRI for these points may be unreliable and hence we excluded these locations from the analysis.

### Thermal nest properties and insulation experiment

To determine thermal characteristics of the nests during the breeding season in 2018, we attached temperature loggers (iButton DS1921Z-F5 and DS1922L, Maxim Integrated Products, Sunnyvale, CA, USA, with resolution 0.125°C and 0.5°C, respectively) to the ceiling of the nest chamber, just above the nest cup (Fig 2A), with the use of poster sticky tabs. We attached the loggers only in the breeding season 2018 due to logistic reasons. The loggers recorded nest chamber air temperature (°C) every hour. If loggers fell off the ceiling or were accidentally detached during a nest check, we reattached them immediately and excluded invalid records due to logger detachment from further analysis. We measured inner nest air chamber temperature in 51 nests that were occupied at the start of the season. We removed loggers if the egg or chick died, or at the end of the season. A summary of logged air temperature in nests is provided in S1 Table.

To assess thermal insulation properties of the nest, we conducted a cooling experiment in 47 nests (with four repetitions per nest). The selected nests were distributed across the whole study area, and most variations of nest lining were equally represented (i.e. from bare rocks or gravel to moss-, grass- or seaweed-insulated). We placed a small (10 ml) glass bottle filled with hot water for 10–15 minutes on the nest chamber floor (Fig 2B). After removing the bottle, we placed a temperature logger (iButton DS1921Z-F5, Maxim Integrated Products, Sunnyvale, CA, USA) on the nest floor to record the temperature of the floor (°C) every minute for a minimum duration of 20 min (Fig 2C). However, we occasionally left the logger in the nest for up to 60 min due to logistic circumstances in the field (please see S1 File for further calculations and a sensitivity analysis of different measurement durations).

In the occupied nests, we performed experiments when the chick was at least 14 days old, to ensure we could safely take the chick out of the nest for the duration of the experiment. To protect the chick from the wind we put it in a cotton bird bag placed in a plastic container with wooden lid, ensuring sufficient air flow. Experiments were conducted during daylight hours.

We could not quantify nest lining properties, such as composition and weight, as this would have required the collection of all nest lining material in the nests. Since Wilson’s storm-petrel breeding pairs might return to the same nets burrow every breeding season, removal of the nest lining could have been detrimental for their breeding success in the next seasons.

### Analyses

We performed all analyses in R software (version 3.4.3) [72]. For calculating the circular mean (mean_circular_) and standard deviation (SD_circular_) of the entrance orientation and nest site orientation, we used the *mean*.*circular* and *sd*.*circular* functions from the *circular* package [73]. To correct for skewness of the data, we log-transformed nest entrance size, nest width and the TRI. We tested the difference between air temperature inside and outside the nest in the breeding season of 2018, using a two sample Student paired *t*-test.

To examine the effects of nest characteristics and weather conditions on the microclimate and breeding parameters, we modelled all relevant parameters and selected the best models based on the delta of the corrected Akaike’s Information Criterion (ΔAICc, calculated using the *dredge* function from the *MuMIn* package) [74]; the use of AICc instead of the regular AIC is recommended in the case of a small ratio between the sample size and the number of modelled parameters [75,76]. A description of the starting models and the selected models is provided below. To estimate the goodness of fit of the models we calculated the likelihood-ratio based pseudo R^2^ (R^2^_p_), [74,77].

If it was not possible to select the best model based on the ΔAICc, due to very similar ΔAIC values in high-ranked models, we performed model averaging of all models within 2 AICc units of the model with the lowest AICc to obtain weighted parameter estimates and the relative importance of each parameter, i.e. the sum of the Akaike weights of all the models with ΔAICc < 2 containing this parameter [76]. We only used significant models for model averaging, hence we determined the significance of the models with the Wald chi-square test, using the *wald*.*test* function from the *aod* package [78]. We calculated the weighted parameter estimates using full-model averaging (i.e. when calculating average parameter estimates, for models not containing the variable of interest the parameter estimate is set to 0), which is recommended in case of high model selection uncertainty [79].

To check for multicollinearity between variables in the starting models, we calculated the variance inflation factor (VIF) using the *vif* function from the *car* package [80]. High collinearity (high VIF) is especially problematic when ecological signals are weak, as high VIF may cause non-significant parameter estimates. Expecting the signal to be weak in our data set, but also aiming to minimize the risk of false positives, we selected VIF < 5 as a threshold, which was enough to ensure a safe and flexible approach in regard to our data set [81]. Subsequently, when VIF > 5, we removed the variable from the model.

As some nests were sampled >1 in one year or sampled in both years (i.e. we possibly repeatedly recorded the same breeding pairs), we corrected for pseudoreplication by treating the nest label (hereafter nest ID) as a random effect. We tested the significance of the random effect by a likelihood ratio test (using the *anova* function [72]), which compared the linear mixed effect model with a similar linear model without the nest ID as a random effect. Comparing the output of the model with and without random effects is a way to test for significance of random factors [82]. To enable this comparison, we fit both models using maximum likelihood.

For small sample sizes, we obtained the significance of the parameters by bootstrapping the model fitting of the specific model, including all averaged parameters (1000 iterations). We analysed the snow blocking and breeding output of both breeding seasons combined, to increase the sample size and enhance the power of the model. Parameters were considered significant if p ≤ 0.050, and considered a trend when p ≤ 0.100.

We analysed the effects of weather conditions and nest characteristics on nest air temperature, snow blocking, hatching success and chick survival following a stepwise approach. In step 1 we aimed to investigate how weather conditions affected the nest air temperature during the breeding season of 2018 and the probability of the nest being blocked by snow in the breeding season of 2017 and 2018. Therefore, we first modelled the nest air temperature with the corresponding weather conditions by fitting a linear mixed effect model, using the *lmer* function from the *lme4* package [83] (model selection in S2 Table). Since the interval between the nest temperature measurements was 60 min and the interval between each of the other measurements was 10 min, we matched the nest air temperature at a specific moment with the corresponding weather conditions with an accuracy of ± 5 min. The averaged models predicting nest air temperature included the following predictors: air temperature, northern and eastern wind direction and wind speed (model selection in S2 Table). Similarly, we modelled the snow blocking status (1 = closed, 0 = open) with the mean environmental predictor variables calculated for the three days preceding the nest check and snow cover at the day of the nest check itself (model selection in S3 Table), using the *glmer* function from the *lme4* package [83]. Even though data from two years were included in this model, we decided that year should not be added as a predictor to this model. Since we expected that inter-annual differences in snow blocking the nest entrance are most likely the result of differences in weather conditions, the model already accounts for inter-annual differences by just testing the effects of weather conditions. The averaged models predicting the probability of snow blocking included the following predictors: air temperature, wind speed, northern and eastern wind direction, snow cover and precipitation. In none of the models, interactions between terms were tested, because we would lose too many degrees of freedom. We added nest ID as a random factor to both models. We obtained the significance of the parameters from the model averaging output (*MuMIn* package) [74].

In step 2 we quantified the nest effect on nest air temperature in the 2018 (hereafter the thermal microclimate) and snow blocking in 2017 and 2018 (hereafter the susceptibility to snow blocking), corrected for weather conditions. To quantify the thermal microclimate, we fit a linear mixed effect model to predict nest air temperature with all meterological parameters obtained from the model averaging described in step 1 (model selection in S2 Table) with nest ID as a random effect. From this model, we obtained the thermal microclimate as the conditional modes (i.e. best linear unbiased predictors [84]) of the random nest ID effects, using the *ranef* function from the *lme4* package [83]. Simultaneously, we quantified the susceptibility to snow blocking as the conditional modes of the random nest ID effect from a binomial mixed effect model that predicted snow blocking of the nest. This model included all meteorological parameters obtained from the model averaging described in step 1 (model selection in S3 Table) with nest ID as a random effect. In both models, the random nest ID effect was significant (p < 0.001).

In step 3 we examined how nest orientation, exposure and insulation (see S1 File for more details on insulation) were related to the thermal microclimate and susceptibility to snow blocking, obtained in step 2. We modelled the thermal microclimate with nest characteristics using a linear model, starting with a model that included all possible nest characteristics (model selection in S4 Table). The parameters of the averaged models were: entrance size and northern nest entrance orientation, nest height, depth and width and insulation (S4 Table and S1 File). To examine snow blocking, we modelled the susceptibility to snow blocking using a linear model and started with a model that included all relevant nest characteristics (model selection in S5 Table). The parameters of averaged models were: entrance size, eastern entrance orientation, TRI, and WEI (S5 Table).

In step 4 we modelled each breeding parameter (hatching success and chick survival) in relation to the thermal microclimate and the susceptibility to snow blocking, as quantified in step 2, using the *glmer* function from the *lme4* package [83]. We included the breeding season in the model, to account for inter-annual differences. We considered hatching as successful (response variable = 1) if one egg hatched, and chick survival as successful (response variable = 1) if the hatched chick survived until fledging (i.e. when it reached the age of 50 days). We also investigated the relationship between the breeding parameters and the nest characteristics. To do so, we initially modelled each breeding output parameter (i.e. hatching success and chick survival) with the nest parameters that were associated with the thermal microclimate and snow blocking as obtained in step 3, and breeding season to account for inter-annual differences (S6 and S7 Tables for the model selection of hatching success and chick survival, respectively). We added nest ID as a random effect.

We examined the relation between chick growth and the nest microclimate in both breeding seasons apart from the stepwise analyses described before. We modelled chick growth (i.e. the daily percentage body mass increase, S2 File) with the mean external weather conditions instead of the nest air temperature, as the nest air temperature measurements were limited to the breeding season 2018 (but see S2 File for additional analyses of chick growth). We used a linear mixed effect model and added nest ID as a random effect (model selection in S8 Table). We calculated the means of the explanatory variables based on records collected during the seven days preceding the nest check, so in general these intervals overlapped. We standardized all numerical parameters. We could not include the mean air temperature and the interaction between breeding season and snow cover in the model, due to multicolinearity (S8 Table for model selection and S2 File for additional analyses of chick growth).

Raw data is provided as supplementary material (S9–S15 Tables).

## Results

### Nest characteristics

During the breeding season of 2018 mean ± SE logged air temperature in the Wilson’s storm-petrel nests was 3.30 ± 0.01°C, being on average 0.89 ± 0.01°C warmer than the outside air temperature of 2.41 ± 0.01°C (p < 0.001, Table 2). We found that nest air temperature was significantly related to weather conditions; higher nest air temperature was associated with higher air temperature and northern winds with low speeds (Table 3). In addition, the microclimate varied considerably among the nests, as indicated by the significant random effect of the nest ID (Table 3). The nest-specific thermal microclimate (i.e. the random effect of nest ID) was best predicted by the nest entrance size, the cooling coefficient, nest height, the WEI and the nest orientation. Nests with a more favourable thermal microclimate (i.e. with higher nest air temperature regardless of weather conditions) had a significantly smaller entrance and a lower cooling coefficient value and a lower nest ceiling, and tended to have an eastern nest site orientation, and to be less exposed to wind (Table 3).

**Table 2:** Description of the studied Wilson’s storm-petrel nests.

**Parameter—Nest orientation**		**N**_**nest**_	**Mean**_**circular**_	**±**	**SD**_**circular**_
	Entrance orientation (°)	82	48.44	±	165.85
	Nest site orientation (°)	107	67.15	±	76.84
**Parameter—Nest dimensions**		**N**_**nest**_	**Mean**	**±**	**SE**
	Entrance size (m^2^ ∙ 10^−4^)	101	63.63	±	5.73
	Height (m ∙ 10^−2^)	69	9.40	±	0.47
	Depth (m ∙ 10^−2^)	70	33.09	±	1.49
	Width (m ∙ 10^−2^)	68	19.51	±	0.75
**Parameter—Nest site micro-topography**		**N**_**nest**_	**Mean**	**±**	**SE**	
	Slope (m elevation m^-1^)	107	0.90	±	0.07
	Terrain Ruggedness Index (m elevation change m^-1^)	107	1.28	±	0.11
	Wind Exposition Index	107	1.06	±	0.01
**Parameter—Nest chamber thermal characteristics in 2018**		**N**_**nest**_	**Mean**	**±**	**SE**
	Temperature (°C), mean logged value	51	3.30	±	0.01
^*^	ΔTemperature_nest-air_ (°C)	51	0.89	±	0.01
	Cooling coefficient	47	0.15	±	0.01

*Significant difference, student paired *t*-test: p < 0.001.

**Table 3:** Effects of weather conditions on the nest air temperature in the breeding season of 2018, snow blocking in the breeding season of 2017 and 2018, and effects of nest characteristics on thermal nest microclimate and susceptibility to snow blocking, corrected for weather conditions.

	Parameter	Estimate^1^	±	SE	Relative importance^2^	p-value^3^
Nest air temperature, N_nest_ = 51, N_per nest_ = 223–2256, linear mixed effect model						
	Intercept	2.130	±	0.012		< 0.001
	**Air temperature**	**0.648**	**±**	**0.002**	**1.00**	**< 0.001**
	**Northern wind direction**	**0.035**	**±**	**0.008**	**1.00**	**< 0.001**
	**Wind speed**	**-0.073**	**±**	**0.002**	**1.00**	**< 0.001**
	Eastern wind direction	-0.002	**±**	0.006	0.33	0.701
^4^	**Random effect: nest ID**	**+**				**< 0.001**
Nest snow blocking, N_nest_ = 123, N_per nest_ = 1–43, binomial mixed effect model						
	Intercept	-4.580	±	0.523		< 0.001
	**Air temperature**	**-0.592**	**±**	**0.078**	**1.00**	**< 0.001**
	**Wind speed**	**3.254**	**±**	**0.813**	**1.00**	**< 0.001**
	**Northern wind direction**	**2.114**	**±**	**0.560**	**1.00**	**< 0.001**
	**Snow cover**	**3.456**	**±**	**0.353**	**1.00**	**< 0.001**
	**Precipitation**	**-1.368**	**±**	**0.535**	**1.00**	**0.011**
	Eastern wind direction	0.133	±	0.318	0.35	0.676
^4^	**Random effect: nest ID**	**+**				**< 0.001**
Thermal nest microclimate (nest ID effect from nest air temperature model), N = 27, linear model						
^5^	Intercept	8.360	±	2.709		< 0.001
^5^	**log Entrance size**	**-0.577**	**±**	**0.163**	**1.00**	**< 0.001**
^5^	**Cooling coefficient**	**-8.057**	**±**	**3.455**	**1.00**	**0.002**
^5^	**Nest height**	**-0.098**	**±**	**0.039**	**1.00**	**0.004**
^5^	**Wind Exposition Index**	**-1.251**	**±**	**2.001**	**0.38**	**0.057**
^5^	**Eastern nest site orientation**	**0.065**	**±**	**0.159**	**0.24**	**0.071**
^5^	Nest depth	0.003	±	0.009	0.16	0.145
^5^	Eastern entrance orientation	0.020	±	0.087	0.10	0.229
Susceptibility to snow blocking (nest ID effect from snow blocking model), N = 68, linear model						
^5^	Intercept	0.588	±	0.998		0.074
^5^	**Eastern entrance orientation**	**-0.124**	**±**	**0.129**	**0.66**	**0.034**
^5^	Wind Exposition Index	-0.354	±	0.813	0.27	0.111
^5^	log Terrain Ruggedness Index	0.017	±	0.058	0.16	0.202
^5^	log Entrance size	-0.013	±	0.045	0.15	0.170
^5^	Eastern nest site orientation	-0.011		0.053	0.13	0.320
^5^	Northern entrance orientation	0.006		0.036	0.07	0.167

Significant predictors and trends are in bold.

^1^ Weighted averages of the parameter estimates were calculated using all models within 2 AICc units of the model with the lowest AICc value (S2, S4 and S5 Tables, respectively). The parameter estimates were calculated using the full-model averaging method [79].

^2^ Parameters are ordered according to their relative importance, i.e. the sum of the Akaike weights of all the models with ΔAICc < 2 containing this parameter [76].

^3^ Significant parameters (p ≤ 0.050) and trends (p ≤ 0.100) are indicated in bold.

^4^ Tested using ANOVA to compare the (binomial) mixed effect model with random effect and a similar (binomial) linear model without random effect, both fitted using maximum likelihood

^5^ P-values were obtained by bootstrapping (1000 iterations) the model fitting of the linear models with all averaged parameters, to account for a small data set.

During nest controls on days with episodes of snowfall (one nest check in 2017 and ten in 2018), we recorded that on average 33.8 ± 9.2% of the nests were blocked by snow. Though snow cover during nest controls has a self-evident effect on the likelihood of snow blocking the nest entrance, the chances were also significantly higher when during the previous three days, air temperature and precipitation were low, wind speed was high and wind direction was northern. In addition, the susceptibility to snow blocking was best predicted by the eastern nest entrance orientation and the WEI. Nests were less likely to be blocked by snow when their entrance was oriented towards the east and when the nest site was more exposed to wind. However, only the effect of eastern entrance orientation was significant (Table 3).

### Breeding output

**Fig 3:**
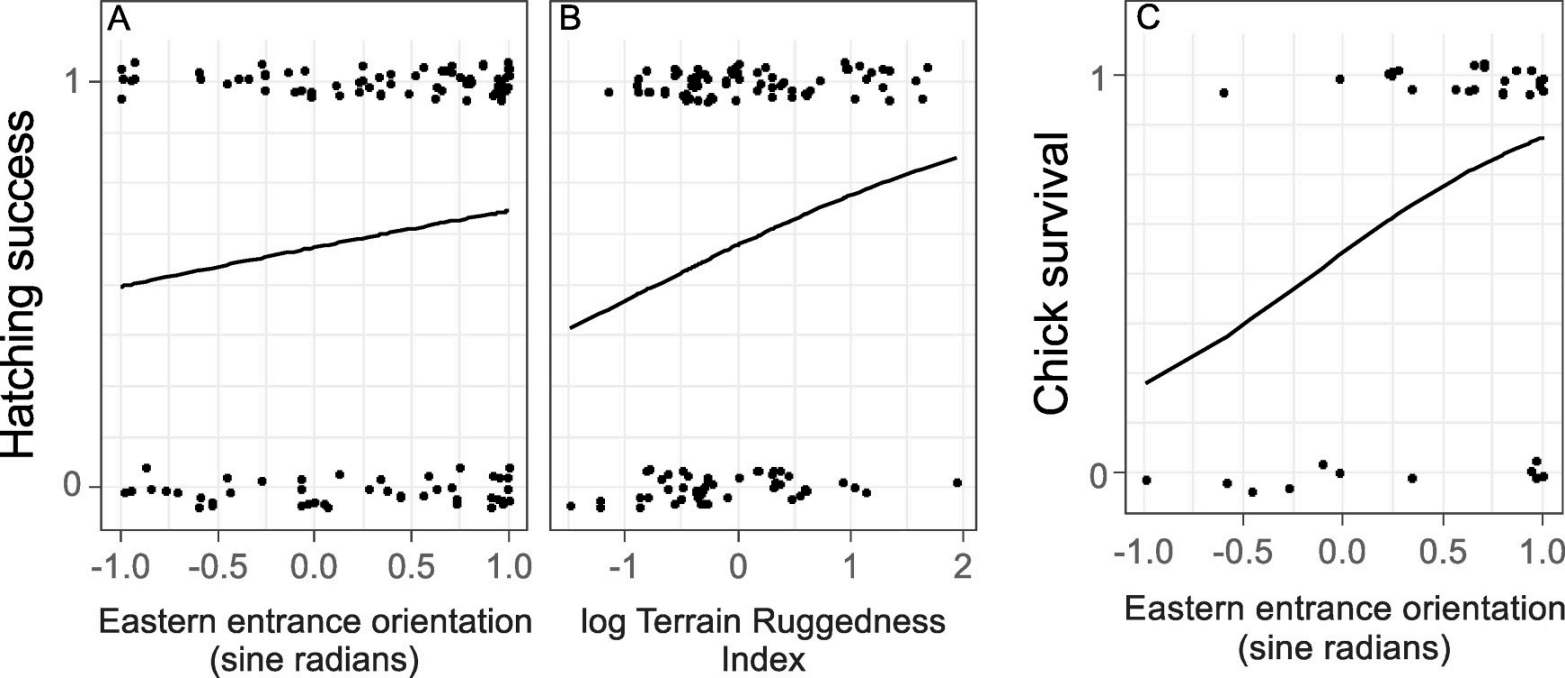
Hatching success and chick survival in response to nest characteristics. The effect of eastern entrance orientation (N = 56, A) and log Terrain Ruggedness Index (N = 56, B) and chick survival in response to eastern entrance orientation (N = 35, C). Only nest characteristics with p ≤ 0.100 are shown. Estimates, p-values and the relative importance of each nest characteristic are provided in Table 4.

Mean hatching success (2017–2018 combined), i.e. the proportion of hatched eggs, was 0.65 ± 0.06 (mean ± SE) and was not affected by the thermal microclimate (p = 0.458, N = 81) or the susceptibility to snow blocking (p = 0.169, N = 81). Still, the best models to predict hatching success included the parameters: eastern entrance orientation, nest height, TRI, breeding season, entrance size, northern entrance orientation, WEI and nest depth (S6 Table). Estimated weighted effects of nest characteristics indicated that hatching success was significantly higher in nests oriented towards the east and with a high TRI, and tended to be higher in 2017 (Fig 3A and 3B and Table 4). The random nest ID effect was not significant (p = 1.000, Table 4), indicating that repeated sampling of the same nest in both years (i.e. the possibility of pseudoreplication due to repeated sampling of the same breeding pairs) did not affect these results.

**Table 4:** Effects of nest characteristics on hatching success and chick survival.

	Parameter	Estimate^1^	±	SE	Relative importance^2^	p-value^3^
Hatching success, N = 56						
	Intercept	-0.671	±	3.818		0.657
	**Eastern entrance orientation**	**1.449**	**±**	**0.579**	**1.00**	**0.008**
	Nest height	0.150	±	0.123	0.82	0.102
	**log Terrain Ruggedness Index**	**0.565**	**±**	**0.716**	**0.56**	**0.050**
	**Breeding season 2018**	**-0.204**	**±**	**0.502**	**0.23**	**0.084**
	log Entrance size	-0.062	±	0.213	0.16	0.367
	Northern entrance orientation	-0.040	±	0.188	0.11	0.298
	Wind Exposition Index	0.595	±	2.696	0.11	0.228
	Nest depth	-0.001		0.009	0.05	0.395
^4^	Random effect: nest ID	+				1.000
Chick survival, N = 35						
	Intercept	2.179	±	2.560		0.344
	**Eastern entrance orientation**	**1.656**	**±**	**0.832**	**1.00**	**0.043**
	Nest depth	-0.033	±	0.044	0.51	0.138
	Eastern nest site orientation	-0.506	±	0.818	0.43	0.166
	Breeding season 2018	-0.185	±	0.561	0.21	0.147
	log Entrance size	-0.087	±	0.295	0.13	0.450
	log Terrain Ruggedness Index	-0.106	±	0.367	0.16	0.215
	Northern entrance orientation	-0.043	±	0.237	0.06	0.471
^4^	Random effect: nest ID	+				1.000

Significant predictors and trends are in bold.

^1^ Weighted averages of the parameter estimates were calculated using all models within 2 AICc units of the model with the lowest AICc value (S6 Table). The parameter estimates were calculated using the full-model averaging method [79].

^2^ Parameters are ordered according to their relative importance, i.e. the sum of the Akaike weights of all the models with ΔAICc < 2 containing this parameter [76].

^**3**^ The significance of the parameters was tested by bootstrapping the model fitting of a model including all averaged parameters. Significant parameters (p ≤ 0.050) and trends (p ≤ 0.100) are indicated in bold.

^4^ Tested using ANOVA to compare the (binomial) mixed effect model with random effect and a similar (binomial) linear model without random effect, both fitted using maximum likelihood

Mean chick survival (2017–2018 combined), i.e. the proportion of hatched chicks that survived until fledging, was 0.69 ± 0.08 (mean ± SE) and was not affected by the thermal microclimate (p = 0.578, N = 42) or the susceptibility to snow blocking (p = 0.896, N = 42). Nevertheless, the best models predicting chick survival included the eastern entrance orientation, nest depth, eastern nest site orientation, breeding season, entrance size, TRI, and northern entrance orientation (S7 Table). The probability of chick survival was significantly higher in nests with an eastern oriented entrance (Fig 3C and Table 4). In addition, estimated weighted effects of nest characteristics indicate that chick survival was affected by nest depth, nest site, and entrance orientation, although these effects were not significant. The random nest ID effect was not significant (p = 1.000, Table 4), indicating that repeated sampling of the same nest in both years (i.e. the possibility of pseudoreplication due to repeated sampling of the same breeding pairs) did not affect these results.

### Chick growth rate

**Fig 4:**
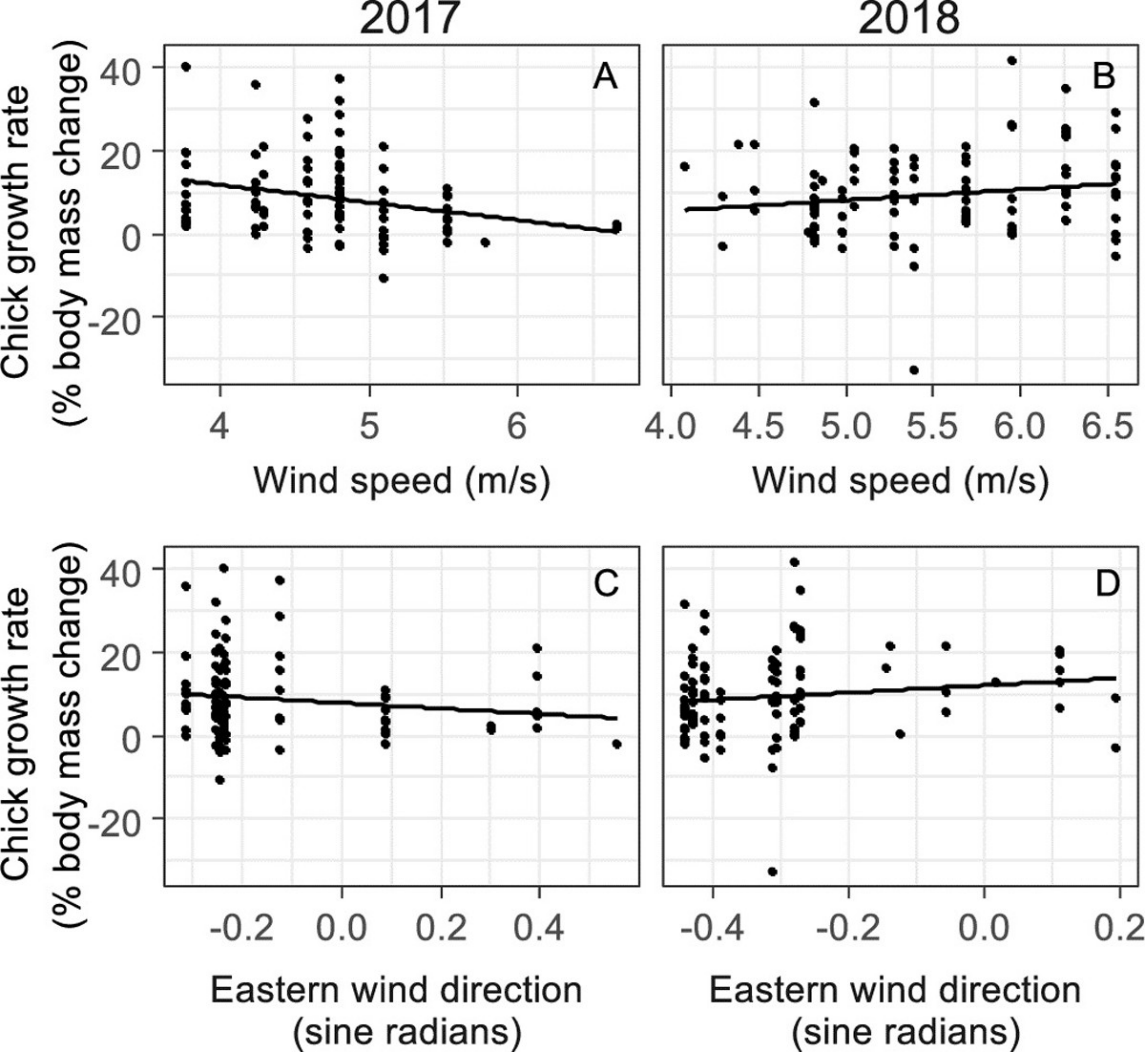
Chick growth rate in response to wind speed in two breeding seasons. The unscaled effect of wind speed and eastern wind direction in the breeding season of 2017 (N = 96, A and C, respectively) and 2018 (N = 100, B and D, respectively). Only parameters with p ≤ 0.100 are shown. Estimates, p-values and the relative importance of each parameter are provided in Table 5.

We found that the mean chick body mass growth rate during the period from 5 to 18 days after hatching in both breeding seasons combined was 12.49 ± 1.02% ∙ day^-1^ (N = 116). The best models to predict the chick growth rate included wind speed, breeding season, snow cover, eastern wind direction, and the interaction between wind speed and breeding season and the interaction between eastern wind direction and breeding season (S8 Table). Chick growth rate was significantly affected by wind speed in both seasons, but the significant interaction between wind speed and breeding season indicates that the effect was different in each season (Fig 4 and Table 5). In addition, the interaction between eastern wind direction and breeding season could be considered a trend, indicated that in each breeding season chick growth tended to be affected differently by an eastern wind direction. Also breeding season, snow cover and eastern wind direction might have independently affected chick growth rate according to the averaged parameter estimates, but none of these effects were significant (Table 5). The random nest ID effect was not significant (p = 1.000), indicating that repeated sampling of the same nest in both years (i.e. the possibility of pseudoreplication due to repeated sampling of the same breeding pairs) did not affect these results.

**Table 5:** Scaled effects of weather conditions and breeding season on the chick growth rate (expressed as % body mass change per day) of chicks between 5 and 18 days old.

	Parameter	Estimate^1^	±	SE	Relative importance^2^	p-value^3^
	Intercept	0.071	**±**	0.013		< 0.001
	**Wind speed**	**-0.033**	**±**	**0.013**	**1.00**	**0.004**
	Breeding season	0.008	**±**	0.019	1.00	0.351
	**Wind speed x Breeding season**	**0.055**	**±**	**0.017**	**1.00**	**< 0.001**
	Snow cover	0.046	**±**	0.059	0.49	0.229
	Eastern wind direction	-0.001	**±**	0.006	0.27	0.308
	**Eastern wind direction x Breeding season**	**0.008**	**±**	**0.016**	**0.27**	**0.069**
^4^	Random effect: nest ID	+				1.000

N_nest_ = 49, N_per nest_ = 2–7, linear mixed effect model. Significant predictors and trends are in bold.

^1^ Weighted averages of the parameter estimates were calculated using all models within 2 AICc units of the model with the lowest AICc value (S6 Table). The parameter estimates were calculated using the full-model averaging method [79].

^2^ Parameters are ordered according to their relative importance, i.e. the sum of the Akaike weights of all the models with ΔAICc < 2 containing this parameter [76].

^3^ The significance of the parameters was tested by bootstrapping the model fitting of a model including all averaged parameters. Significant effects (p ≤ 0.050) and trends (p ≤ 0.100) are indicated in bold.

^4^ Tested using ANOVA to compare the (binomial) mixed effect model with random effect and a similar (binomial) linear model without random effect, both fitted using maximum likelihood

## Discussion

The findings of our study support the hypothesis that under extreme weather conditions of the Antarctic summer, nest burrow characteristics—in particular the nest dimensions, entrance orientation and nest insulation—can provide shelter against the weather and thereby establish a thermally favourable microclimate and protection against snow blocking. In addition, the hypotheses that hatching success and chick survival are higher in nests with characteristics which improve the thermal microclimate and reduce the chance of snow blocking, was supported. However, it remains uncertain whether the observed effects on hatching success and chick survival were through nest air temperature or snow blocking, since the nest parameters that affected breeding output were associated with the thermal microclimate as well as the susceptibility to snow blocking. In addition, the hypotheses that chick growth was enhanced by a high nest air temperature was not supported, as we were unable to detect an unambiguous effect of weather conditions related to nest air temperature on chick growth. Since the effect of nest air temperature on chick growth remains indistinct, it is likely that also breeding output was less affected by the thermal microclimate and the susceptibility to snow blocking was possibly more important. Hence, despite some effects being statistically insignificant, apparent trends add some evidence to a growing awareness of the importance of nest shelter in establishing a thermally favourable microclimate and protection against snow blocking and thereby enhancing breeding success [11–14,20,46,85–87].

Our hypothesis that hatching success and chick survival would be favoured in nests with nest characteristics which improve the thermal microclimate and reduce the chance of snow blocking, was supported, as hatching success as well as chick survival was positively affected by an eastern nest entrance orientation. However, it remains uncertain if the observed effect of nest characteristics originated from the thermal microclimate or from the susceptibility to snow blocking, since an eastern nest orientation (either of the nest site or the nest entrance, respectively) was associated with a more favourable nest specific microclimate as well as with a lower susceptibility to snow blocking. The positive effect of an eastern nest orientation regarding the thermal microclimate and the susceptibility to snow blocking, could be explained by the weather conditions at KGI. First of all, the predominant wind direction during the breeding seasons of 2017 and 2018 ranged from north to west to southwest [61]. In addition, eastern wind was associated with higher air temperatures and less precipitation (i.e. snow or rain) [88]. Therefore, it is likely that by selecting nests oriented towards the east, parents select nests sheltered in terms of the thermal microclimate and snow blocking. This is underlined by the mean orientation of the nest entrance (38.44° i.e. north-east) and nest site (67.15° i.e. east north-east) obtained in this study and our observation that the hourly mean nest air temperature was significantly higher than the hourly mean ambient air temperature during the breeding season of 2018. Although a difference of 0.9°C between the outside air temperature and nest air temperature might seem small, in the harsh climate of the sub-Antarctic and temperatures close to 0°C, such a small difference could be critical for breeding success. In addition, the importance of the thermal microclimate of the nest to hatching success and chick survival has also been reported in other studies, as it could hamper the embryonic development, increase the energetic demands of the incubating parents and reduce the immune response of chicks [22–30,89]. However, the ambiguous effects of weather conditions related to nest air temperature on chick growth, do indicate that the thermal microclimate of the nest is not of major importance for the development of the Wilson’s storm-petrel chicks. Therefore, the observed effects of nest characteristics on the breeding output are possibly mainly through the susceptibility to snow blocking. Indeed, snow blocking of the nest has been reported in several studies as a major cause of egg and chick mortality in Wilson’s storm-petrels [43,44,53,57,58]. Similar results have been obtained in a study at the Windmill Islands, East Antarctic, where Wilson’s storm-petrel nests were limited by the snow accumulation patterns during the breeding season [47]. Hence, despite the absence of an observed direct effect of the thermal microclimate and susceptibility to snow blocking on the breeding success, the effect of an eastern nest orientation indicates that the thermal microclimate and the susceptibility to snow blocking could both be an important factor determining breeding success. Moreover, they could serve as an important driver of nest site selection, despite the highly variable and rather unpredictable nature of the Antarctic weather conditions and snow accumulation patterns in space and time [90–93].

Effects of weather conditions on chick growth rate were complex, as both the effect of wind speed and wind direction depended on the breeding season. In 2017, wind speed and eastern wind direction negatively affected chick growth, while in 2018 chick growth was enhanced by higher wind speeds and an eastern wind direction. On the one hand, the negative effect of wind speed in 2017 and the positive effect of eastern wind direction in 2018 support our expectation that a higher nest air temperature favours chick growth. Firstly, due to the negative effect of wind speed on nest air temperature due to wind chilling effect, observed in this study. Secondly, due to the association of eastern wind with higher ambient air temperatures, reported in the study of Kejna and Laska [88]. Hence, it appears that higher nest burrow air temperatures and ambient air temperatures favour chick growth, which is in accordance with the study of Wasilewski [57] performed in the same area, in which nest burrow air temperature was positively related to chick growth rate. On the other hand, the opposite effects of eastern wind direction and wind speed in the two study periods might indicate that nest temperature is not a major driver of chick growth in Wilson’s storm-petrel chicks, especially, when also the results of the additional analyses of chick growth in S2 File are considered (i.e. chick growth tends to be negatively affected by nest air temperature and the chick growth parameter is positively affected by nest height). Indeed, birds breeding at high latitudes or altitudes are typically resilient to the harsh climate of these areas [34]. Also, Wilson’s storm-petrel chicks are known to have developed several strategies to cope with the harsh and unpredictable weather, such as a relatively high resting metabolism to maintain thermoregulation and the ability to survive and even induce facultative hypothermia during extreme circumstances (i.e. torpor) [44,51,52,57]. Some of these adaptations are, however, energetically demanding and hence, food provisioning is thought to be one of the main limitations of chick growth and breeding success [43,51,63]. Additionally, studies on common terns (*Sterna hirundo*) breeding in Europe, and Antarctic petrels (*Thalassoica antarctica*) have revealed that susceptibility of chicks to bad weather depends on their body condition [94,95]. Therefore, the opposing effects of weather conditions in the two study years might be explained by differences in food availability between 2017 and 2018, and between periods with certain weather conditions [43,58,96–99]. The slower chick growth in 2017 may indicate a relatively low food availability, which could have reinforced the negative response of chick growth to the low nest air temperature caused by the wind chill effects of the strong winds. Higher food availability in 2018 could make the chicks more resilient to low nest air temperatures, and strong eastern winds could even have a positive impact on chick growth by enhancing food availability due to vertical mixing of Antarctic ocean water [96–99] and due to higher air temperatures associated with eastern wind [88]. Therefore, the effect of the thermal microclimate on chick growth is probably only apparent when food supply is limited.

In addition to nest characteristics, parental effort and quality are likely to affect the breeding success and chick growth, however, we did not control for these variables. Therefore, it should be taken into account that the observed response of the breeding output to nest characteristics, could also be attributed to occupation of better quality nest burrows by higher quality parents. In that case, the observed effects are possibly not solely induced by the effects of the thermal microclimate or snow blocking, but could be (partly) ascribed to the quality of the parents. In fact, in common eiders breeding in the Arctic, the observed reduced breeding success at unfavourable nest sites was attributed to the lower quality of the parents occupying these nests, rather than to the unfavourable microclimate of these nests [48]. Moreover, the effects of weather conditions on chick growth could also be attributed to the parental effort under certain weather conditions rather than directly onto the weather conditions. Indeed, unfavourable environmental circumstances are likely to reduce the parental effort in long living seabirds, although this response varies greatly between individuals [43,51,63]. Nevertheless, despite the presumably complex effects of parental care and quality, we were able to detect some effects of nest characteristics and weather conditions that can, to a certain extent, be linked to the thermal microclimate and the susceptibility to snow blocking of the nest.

Finally, in the Antarctic, the effects of the expected increase in greenhouses gases over the next century, will be substantial because of the speed at which atmospheric greenhouse gas concentrations rise, and because of polar amplification of the global warming signal [100]. In the next 100 years, mean Antarctic surface temperatures are projected to increase 0.34°C per decade and annual snowfall will increase by 20% in total [100]. Nest characteristics that determine the chance of snow blocking are therefore likely to become more important for breeding success. At a more regional scale, the natural highly variable climatic conditions of the Antarctic Peninsula resulted in a significant decrease in mean annual air surface temperature in the previous decade, which was especially apparent in the northwest of the Peninsula and on the South Shetland Islands [101,102]. Hence, it is likely that, despite global warming, such an unpredictable climate will keep demanding thermally favourable nest burrows, to support the breeding success.

Our study indicates the importance of nest characteristics like nest dimensions, insulation and orientation in establishing a thermally favourable microclimate and limiting snow blocking for the Wilson’s storm-petrel nests. However, the response of hatching success, chick growth and chick survival to the nest characteristics, was complex and probably greatly affected by other parameters, such as food availability, and parental effort and quality. This might explain why the effect of weather conditions through nest air temperature on chick growth remained indistinct. Nevertheless, nest characteristics that enhanced the thermal microclimate and reduced the risk of snow blocking positively affected both hatching success and chick survival. The projected increase in snowfall, due to climate change, will probably enhance the importance of nest characteristics that determine snow blocking. Additionally, the highly variable nature of the Antarctic climate will probably keep demanding for thermally favourable nest burrows. Thus, our study shows the importance of nest characteristics in enhancing the breeding output and chick growth of the burrow-nesting seabirds breeding in harsh polar conditions.

## Supporting information

S1 Table Summary of logged air temperature in nests. Nest ID, number of records (N), the first and last date of temperature logged and the mean nest air temperature ± SE.
(PDF)

S2 Table Model selection for the effect of weather conditions on nest air temperature. Unscaled parameter estimates for each model are shown. Models used in model averaging are indicated in bold. Model used for extracting the random nest effect is marked with an asterisk.
(PDF)

S3 Table Model selection for the effect of weather conditions during the previous three days and snow cover during the nest check on snow blocking of the nest. Unscaled parameter estimates for each model are shown. Only models within 30 units of AICc are shown, due to the high number of possible models. Models used in model averaging are indicated in bold. Model used for extracting the random nest effect is marked with an asterisk.
(PDF)

S4 Table Model selection for the effect of nest characteristics on the thermal microclimate. Unscaled parameter estimates for each model are shown. Only models within 7 units of AICc are shown, due to the high number of possible models. Models used in model averaging are indicated in bold.
(PDF)

S5 Table Model selection for the effect of nest characteristics on the susceptibility to snow blocking. Unscaled parameter estimates for each model are shown. Only models within 7 units of AICc are shown, due to the high number of possible models. Models used in model averaging are indicated in bold.
(PDF)

S6 Table Model selection for the effects of nest parameters on hatching success. Unscaled parameter estimates for each model are shown. Only models within 3 units of AICc are shown, due to the high number of possible models. Models used in model averaging are indicated in bold.
(PDF)

S7 Table Model selection for the effects of nest parameters on chick survival. Unscaled parameter estimates for each model are shown. Only models within 4 units of AICc are shown, due to the high number of possible models. Models used in model averaging are indicated in bold.
(PDF)

S8 Table Model selection for the effects of weather conditions and breeding season on chick growth rate. Chick growth rate was measured as the % body mass change. Scaled parameter estimates for each model are shown. Models used in model averaging are indicated in bold.
(PDF)

S9 Table Air temperature, wind speed and wind direction data. (CSV)

S10 Table Snow cover and precipitation data. (CSV)

S11 Table Nest air temperature data. (CSV)

S12 Table Snow blocking data. (CSV)

S13 Table Micro-topography and cooling coefficient data. (CSV)

S14 Table Chick growth data. (CSV)

S15 Table Breeding output and growth parameter data. (CSV)

S1 File Additional information on nest insulation. The quantification of the nest insulation and a sensitivity analysis of different measurement durations.
(PDF)

S2 File Additional analyses of chick growth. The calculations of chick growth, the effect of nest air temperature on chick growth in 2018 and the effect of nest characteristics on chick growth.
(PDF)
