# Supplementary material for: Nest characteristics determine nest microclimate and affect breeding output in an Antarctic seabird, the Wilson’s storm-petrel
Source: PLoS One. 2019 Jun 13;14(6):e0217708. doi: 10.1371/journal.pone.0217708 (PMC6564424; doi:10.1371/journal.pone.0217708)
Supplement: S1 Table — Nest ID, number of records (N), the first and last date of temperature logged and the mean nest air temperature ± SE. (PDF) [file pone.0217708.s001.pdf]

S1 Table. Summary of logged air temperature in nests. Nest ID, number of records (N), the first and last date of temperature logged and the mean nest air temperature ± SE.

Nest ID	N	Date first record	Date last record	Mean nest air temperature (°C)	±	SE
1709	1518	26/01/2018	30/03/2018	3.3	±	0.05
1711	1128	20/01/2018	07/03/2018	4.8	±	0.05
1712	1295	20/01/2018	14/03/2018	4.0	±	0.06
1713	1317	20/01/2018	15/03/2018	4.4	±	0.06
1714	967	26/01/2018	07/03/2018	3.1	±	0.06
1716	1747	20/01/2018	02/04/2018	3.7	±	0.04
1719	1638	21/01/2018	30/03/2018	2.6	±	0.06
1720	1128	20/01/2018	12/03/2018	4.4	±	0.05
1723	964	26/01/2018	07/03/2018	4.0	±	0.05
1724	1638	21/01/2018	30/03/2018	2.4	±	0.04
1725	1087	21/01/2018	07/03/2018	2.7	±	0.04
1727	223	26/01/2018	04/02/2018	4.4	±	0.10
1730	439	23/01/2018	10/02/2018	5.2	±	0.06
1736	775	23/01/2018	24/02/2018	5.7	±	0.07
1740	1559	22/01/2018	03/04/2018	2.4	±	0.03
1742	459	23/01/2018	11/02/2018	3.3	±	0.09
1743	463	23/01/2018	11/02/2018	5.0	±	0.10
1744	290	23/01/2018	04/02/2018	3.0	±	0.11
1746	1586	23/01/2018	30/03/2018	3.6	±	0.04
1749	1607	23/01/2018	30/03/2018	3.5	±	0.07
1751	1135	26/01/2018	14/03/2018	2.7	±	0.06
1753	1590	26/01/2018	02/04/2018	2.7	±	0.05
1754	1638	21/01/2018	30/03/2018	2.6	±	0.06
1755	1729	21/01/2018	03/04/2018	4.2	±	0.05

1758	463	26/01/2018	14/02/2018	2.9	±	0.08
1759	1105	21/01/2018	08/03/2018	2.7	±	0.06
1761	1726	21/01/2018	03/04/2018	2.0	±	0.04
1763	1638	21/01/2018	30/03/2018	2.9	±	0.03
1764	1414	23/01/2018	23/03/2018	3.7	±	0.06
1766	964	21/01/2018	02/03/2018	4.6	±	0.04
1767	1679	20/01/2018	30/03/2018	3.3	±	0.04
1768	2256	20/01/2018	07/03/2018	3.2	±	0.03
1801A	1368	24/01/2018	22/03/2018	2.6	±	0.07
1801K	1590	26/01/2018	02/04/2018	4.1	±	0.08
1801R	1518	26/01/2018	30/03/2018	3.0	±	0.05
1803A	1084	05/02/2018	22/03/2018	2.9	±	0.08
1803R	1584	26/01/2018	02/04/2018	2.6	±	0.07
1805A	1703	23/01/2018	03/04/2018	2.6	±	0.04
1806D	248	10/02/2018	20/02/2018	3.5	±	0.05
1806K	868	10/02/2018	23/03/2018	3.2	±	0.08
1807D	727	28/01/2018	27/02/2018	4.6	±	0.05
1808D	1246	29/01/2018	22/03/2018	4.5	±	0.07
1809K	1614	26/01/2018	03/04/2018	3.4	±	0.07
1810K	1638	21/01/2018	30/03/2018	2.2	±	0.05
1811K	1638	21/01/2018	30/03/2018	2.7	±	0.05
1811R	1008	19/02/2018	02/04/2018	2.6	±	0.09
1812K	1298	26/01/2018	21/03/2018	3.4	±	0.07
1813K	1577	20/01/2018	02/04/2018	4.2	±	0.04
1813R	1008	19/02/2018	02/04/2018	3.5	±	0.08
1814R	507	12/03/2018	02/04/2018	3.4	±	0.06
1816R	510	13/03/2018	03/04/2018	2.2	±	0.11
