# Supplementary material for: Nest characteristics determine nest microclimate and affect breeding output in an Antarctic seabird, the Wilson’s storm-petrel
Source: PLoS One. 2019 Jun 13;14(6):e0217708. doi: 10.1371/journal.pone.0217708 (PMC6564424; doi:10.1371/journal.pone.0217708)
Supplement: S2 Table — Unscaled parameter estimates for each model are shown. Models used in model averaging are indicated in bold. Model used for extracting the random nest effect is marked with an asterisk. (PDF) [file pone.0217708.s002.pdf]

S2 Table. Model selection for the effect of weather conditions on nest air temperature. Unscaled parameter estimates for each model are shown. Models used in model averaging are indicated in bold. Model used for extracting the random nest effect is marked with an asterisk.

	**Intercept**	**Air temperature**	**Northern wind direction**	**Eastern wind direction**	**Wind speed**	**R^2_p**	**ΔAICc**
	**2.130**	**0.648**	**0.035**	**-**	**-0.073**	**0.557**	**0.00**
*	**2.130**	**0.648**	**0.035**	**-0.007**	**-0.073**	**0.557**	**1.37**
	2.130	0.648	-	-	-0.073	0.557	15.39
	2.130	0.648	-	-0.006	-0.073	0.557	16.85
	1.759	0.639	0.037	-	-	0.544	1759.56
	1.759	0.639	0.037	-0.010	-	0.544	1760.28
	1.759	0.639	-	-	-	0.544	1776.46
	1.759	0.639	-	-0.009	-	0.544	1777.30
	3.450	-	0.071	0.020	-0.028	0.003	49295.73
	3.450	-	0.072	-	-0.028	0.002	49296.41
	3.450	-	-	0.021	-0.028	0.002	49326.70
	3.450	-	-	-	-0.028	0.002	49327.64
	3.300	-	0.072	0.019	-	0.001	49411.09
	3.300	-	0.072	-	-	0.001	49411.44
	3.301	-	-	0.020	-	0.000	49442.54
	3.301	-	-	-	-	0.000	49443.13
