# Supplementary material for: Nest characteristics determine nest microclimate and affect breeding output in an Antarctic seabird, the Wilson’s storm-petrel
Source: PLoS One. 2019 Jun 13;14(6):e0217708. doi: 10.1371/journal.pone.0217708 (PMC6564424; doi:10.1371/journal.pone.0217708)
Supplement: S3 Table — Unscaled parameter estimates for each model are shown. Only models within 30 units of AICc are shown, due to the high number of possible models. Models used in model averaging are indicated in bold. Model used for extracting the random nest effect is marked with an asterisk. (PDF) [file pone.0217708.s003.pdf]

S3 Table. Model selection for the effect of weather conditions during the previous three days and snow cover during the nest check on snow blocking of the nest. Unscaled parameter estimates for each model are shown. Only models within 30 units of AICc are shown, due to the high number of possible models. Models used in model averaging are indicated in bold. Model used for extracting the random nest effect is marked with an asterisk.

	Intercept	Air temp	Northern wind direction	Eastern wind direction	Precipitation	Wind speed	Snow cover	R^2_p	ΔAICc
	**-4.594**	**-0.601**	**2.045**	**-**	**-1.343**	**3.290**	**3.469**	**0.507**	**0.00**
*	**-4.552**	**-0.575**	**2.243**	**0.381**	**-1.417**	**3.185**	**3.431**	**0.508**	**1.26**
	-4.894	-0.662	1.916	-	-	2.659	2.909	0.501	4.46
	-4.866	-0.649	2.033	0.214	-	2.565	2.864	0.502	6.24
	-3.118	-0.568	2.243	0.735	-0.924	-	3.115	0.496	12.33
	-3.066	-0.606	1.944	-	-0.775	-	3.158	0.494	13.04
	-3.379	-0.654	1.904	-	-	-	2.779	0.492	13.40
	-4.818	-0.504	-	-	-1.144	3.260	3.535	0.494	13.42
	-3.466	-0.627	2.166	0.602	-	-	2.678	0.493	13.53
	-4.917	-0.540	-	-0.350	-1.071	3.451	3.581	0.494	14.60
	-5.104	-0.563	-	-	-	2.760	3.067	0.489	16.40
	-5.228	-0.607	-	-0.471	-	3.073	3.177	0.491	16.89
	-3.545	-0.577	-	-	-	-	2.947	0.480	24.43
	-3.259	-0.533	-	-	-0.687	-	3.274	0.482	24.46
	-3.540	-0.580	-	-0.036	-	-	2.954	0.480	26.43

	-3.262	-0.528	-	0.065	-0.702	-	3.269	0.482	26.44
