# Supplementary material for: Nest characteristics determine nest microclimate and affect breeding output in an Antarctic seabird, the Wilson’s storm-petrel
Source: PLoS One. 2019 Jun 13;14(6):e0217708. doi: 10.1371/journal.pone.0217708 (PMC6564424; doi:10.1371/journal.pone.0217708)
Supplement: S4 Table — Unscaled parameter estimates for each model are shown. Only models within 7 units of AICc are shown, due to the high number of possible models. Models used in model averaging are indicated in bold. (PDF) [file pone.0217708.s004.pdf]

S4 Table. Model selection for the effect of nest characteristics on the thermal microclimate. Unscaled parameter estimates for each model are shown. Only models within 7 units of AICc are shown, due to the high number of possible models. Models used in model averaging are indicated in bold.

**Intercept**	**log Entrance size**	**Cooling coefficient**	**Nest height**	**Northern entrance orientation**	**Eastern entrance orientation**	**Northern nest site orientation**	**Eastern nest site orientation**	**Nest depth**	**log TRI**	**WEI**	**log Nest width**	**R^2_p**	**ΔAICc**
**6.775**	**-0.538**	**-7.598**	**-0.100**	**-**	**-**	**-**	**-**	**-**	**-**	**-**	**-**	**0.586**	**0.00**
**10.288**	**-0.555**	**-8.387**	**-0.092**	**-**	**-**	**-**	**-**	**-**	**-**	**-3.124**	**-**	**0.644**	**0.09**
**7.198**	**-0.654**	**-7.438**	**-0.106**	**-**	**-**	**-**	**-**	**0.019**	**-**	**-**	**-**	**0.629**	**0.98**
**11.185**	**-0.615**	**-9.371**	**-0.090**	**-**	**-**	**-**	**0.295**	**-**	**-**	**-3.497**	**-**	**0.684**	**1.24**
**7.164**	**-0.586**	**-8.323**	**-0.099**	**-**	**-**	**-**	**0.240**	**-**	**-**	**-**	**-**	**0.614**	**1.85**
**6.836**	**-0.551**	**-7.305**	**-0.105**	**-**	**0.204**	**-**	**-**	**-**	**-**	**-**	**-**	**0.612**	**1.92**
10.194	-0.647	-8.157	-0.098	-	-	-	-	0.015	-	-2.732	-	0.671	2.08
7.357	-0.693	-7.030	-0.114	-	0.263	-	-	0.022	-	-	-	0.670	2.14
7.708	-0.724	-8.274	-0.105	-	-	-	0.283	0.021	-	-	-	0.666	2.40
6.761	-0.534	-7.618	-0.101	0.136	-	-	-	-	-	-	-	0.600	2.60
5.349	-0.481	-	-0.114	-	-	-	-	-	-	-	-	0.466	2.74
11.167	-0.727	-9.208	-0.096	-	-	-	0.324	0.018	-	-3.087	-	0.719	2.89
10.017	-0.563	-8.093	-0.097	-	0.156	-	-	-	-	-2.841	-	0.658	2.90
10.255	-0.550	-8.402	-0.094	0.133	-	-	-	-	-	-3.107	-	0.657	2.98
10.048	-0.516	-7.693	-0.085	-	-	0.151	-	-	-	-3.377	-	0.656	3.02
5.966	-0.505	-7.794	-0.099	-	-	-	-	-	-	-	0.192	0.592	3.04
6.455	-0.514	-7.135	-0.096	-	-	0.092	-	-	-	-	-	0.591	3.08

6.779	-0.539	-7.592	-0.100	-	-	-	-	-	-0.008	-	-	0.586	3.34
7.218	-0.659	-7.448	-0.108	0.169	-	-	-	0.020	-	-	-	0.649	3.43
5.829	-0.605	-	-0.120	-	-	-	-	0.020	-	-	-	0.514	3.64
10.988	-0.569	-8.541	-0.080	-	-	0.215	0.342	-	-	-3.917	-	0.708	3.69
10.298	-0.554	-8.410	-0.091	-	-	-	-	-	0.021	-3.143	-	0.644	3.77
10.096	-0.549	-8.410	-0.092	-	-	-	-	-	-	-3.079	0.034	0.644	3.78
10.398	-0.576	-9.847	-	-	-	-	-	-	-	-3.734	-	0.510	3.80
5.485	-0.499	-	-0.120	-	0.239	-	-	-	-	-	-	0.502	4.19
7.920	-0.489	-	-0.109	-	-	-	-	-	-	-2.387	-	0.500	4.26
6.824	-0.628	-6.877	-0.101	-	-	0.111	-	0.019	-	-	-	0.636	4.26
6.096	-0.653	-	-0.129	-	0.301	-	-	0.024	-	-	-	0.568	4.31
6.140	-0.558	-9.037	-	-	-	-	-	-	-	-	-	0.427	4.37
9.796	-0.681	-7.697	-0.106	-	0.215	-	-	0.019	-	-2.251	-	0.697	4.42
6.525	-0.625	-7.602	-0.105	-	-	-	-	0.018	-	-	0.158	0.632	4.45
4.810	-0.432	-	-0.104	-	-	0.213	-	-	-	-	-	0.494	4.54
7.144	-0.588	-7.957	-0.103	-	0.166	-	0.197	-	-	-	-	0.630	4.61
7.749	-0.744	-7.779	-0.112	-	0.221	-	0.232	0.024	-	-	-	0.694	4.66
7.197	-0.654	-7.439	-0.105	-	-	-	-	0.019	0.002	-	-	0.629	4.67
11.366	-0.641	-10.872	-	-	-	-	0.319	-	-	-4.121	-	0.557	4.85
10.144	-0.652	-8.150	-0.101	0.161	-	-	-	0.017	-	-2.669	-	0.690	4.94
6.738	-0.555	-7.723	-0.093	-	-	0.134	0.266	-	-	-	-	0.623	4.97
