# Supplementary material for: Nest characteristics determine nest microclimate and affect breeding output in an Antarctic seabird, the Wilson’s storm-petrel
Source: PLoS One. 2019 Jun 13;14(6):e0217708. doi: 10.1371/journal.pone.0217708 (PMC6564424; doi:10.1371/journal.pone.0217708)
Supplement: S5 Table — Unscaled parameter estimates for each model are shown. Only models within 7 units of AICc are shown, due to the high number of possible models. Models used in model averaging are indicated in bold. (PDF) [file pone.0217708.s005.pdf]

S5 Table. Model selection for the effect of nest characteristics on the susceptibility to snow blocking. Unscaled parameter estimates for each model are shown. Only models within 7 units of AICc are shown, due to the high number of possible models. Models used in model averaging are indicated in bold.

**Intercept**	**log Entrance size**	**Northern entrance orientation**	**Eastern entrance orientation**	**Northern nest site orientation**	**Eastern nest site orientation**	**log TRI**	**WEI**	**R^2_p**	**ΔAICc**
**0.113**	**-**	**-**	**-0.190**	**-**	**-**	**-**	**-**	**0.050**	**0.00**
**0.072**	**-**	**-**	**-**	**-**	**-**	**-**	**-**	**0.000**	**0.75**
**1.467**	**-**	**-**	**-0.186**	**-**	**-**	**-**	**-1.266**	**0.074**	**0.78**
**1.494**	**-**	**-**	**-**	**-**	**-**	**-**	**-1.329**	**0.027**	**1.38**
**0.758**	**-0.077**	**-**	**-0.199**	**-**	**-**	**-**	**-**	**0.064**	**1.38**
**0.110**	**-**	**-**	**-0.174**	**-**	**-**	**0.095**	**-**	**0.064**	**1.40**
**0.072**	**-**	**-**	**-**	**-**	**-**	**0.120**	**-**	**0.023**	**1.59**
**0.111**	**-**	**0.079**	**-0.204**	**-**	**-**	**-**	**-**	**0.059**	**1.70**
**2.404**	**-0.091**	**-**	**-0.195**	**-**	**-**	**-**	**-1.423**	**0.095**	**1.85**
**0.115**	**-**	**-**	**-**	**-**	**-0.116**	**-**	**-**	**0.017**	**1.93**
**0.132**	**-**	**-**	**-0.172**	**-**	**-0.061**	**-**	**-**	**0.054**	**2.00**
0.109	-	-	-0.187	0.027	-	-	-	0.051	2.20
1.447	-	-	-0.170	-	-	0.093	-1.251	0.088	2.28
1.466	-	-	-	-	-	0.117	-1.303	0.049	2.32
0.613	-0.064	-	-	-	-	-	-	0.010	2.34
1.548	-	0.091	-0.201	-	-	-	-1.344	0.086	2.36

2.315	-0.080	-	-	-	-	-	-1.468	0.042	2.70
1.474	-	-	-	-	-0.108	-	-1.273	0.042	2.74
0.069	-	0.047	-	-	-	-	-	0.003	2.75
0.066	-	-	-	0.048	-	-	-	0.003	2.76
0.107	-	0.099	-0.189	-	-	0.111	-	0.078	2.87
1.459	-	-	-0.169	-	-0.054	-	-1.244	0.078	2.90
0.725	-0.073	-	-0.183	-	-	0.090	-	0.077	2.93
0.109	-	-	-	-	-0.101	0.109	-	0.036	3.08
1.453	-	-	-0.184	0.019	-	-	-1.256	0.075	3.09
0.742	-0.075	0.076	-0.212	-	-	-	-	0.073	3.18
0.585	-0.061	-	-	-	-	0.117	-	0.032	3.30
1.548	-	0.059	-	-	-	-	-1.383	0.032	3.32
0.067	-	0.073	-	-	-	0.133	-	0.031	3.38
2.348	-0.088	-	-0.180	-	-	0.087	-1.402	0.106	3.51
1.464	-	-	-	0.039	-	-	-1.306	0.029	3.52
2.471	-0.090	0.089	-0.210	-	-	-	-1.497	0.106	3.53
0.745	-0.073	-	-0.183	-	-0.051	-	-	0.067	3.53
0.126	-	-	-0.159	-	-0.053	0.091	-	0.067	3.54
1.542	-	0.110	-0.186	-	-	0.111	-1.343	0.105	3.59
0.064	-	-	-	0.056	-	0.123	-	0.027	3.61
0.104	-	-	-0.170	0.035	-	0.097	-	0.066	3.64

0.609	-0.059	-	-	-	-0.111	-	-	0.026	3.68
0.755	-0.076	-	-0.199	0.002	-	-	-	0.064	3.71
0.131	-	0.082	-0.185	-	-0.065	-	-	0.064	3.73
2.252	-0.076	-	-	-	-	0.113	-1.437	0.063	3.78
0.113	-	0.057	-	-	-0.122	-	-	0.022	3.89
1.451	-	-	-	-	-0.094	0.107	-1.257	0.060	3.98
1.541	-	0.086	-	-	-	0.133	-1.378	0.060	4.00
0.110	-	0.077	-0.203	0.011	-	-	-	0.059	4.02
0.108	-	-	-	0.035	-0.112	-	-	0.019	4.09
2.368	-0.088	-	-0.183	-	-0.041	-	-1.401	0.096	4.14
2.439	-0.094	-	-0.197	-0.014	-	-	-1.434	0.095	4.25
2.240	-0.075	-	-	-	-0.101	-	-1.407	0.055	4.26
0.128	-	-	-0.170	0.022	-0.059	-	-	0.055	4.29
0.599	-0.063	0.043	-	-	-	-	-	0.013	4.43
1.429	-	-	-	0.047	-	0.120	-1.275	0.052	4.48
1.542	-	0.093	-0.184	-	-0.059	-	-1.322	0.090	4.52
0.699	-0.070	0.095	-0.197	-	-	0.106	-	0.090	4.53
1.441	-	-	-0.157	-	-0.046	0.090	-1.233	0.090	4.53
0.569	-0.060	-	-	0.029	-	-	-	0.011	4.54
1.427	-	-	-0.167	0.027	-	0.095	-1.236	0.089	4.63
1.536	-	0.069	-	-	-0.115	-	-1.332	0.049	4.64

2.353	-0.079	0.056	-	-	-	-	-1.518	0.047	4.75
1.548	-	0.091	-0.201	0.000	-	-	-1.344	0.086	4.77
0.106	-	0.080	-	-	-0.107	0.123	-	0.046	4.85
0.064	-	0.041	-	0.041	-	-	-	0.005	4.88
0.584	-0.057	-	-	-	-0.096	0.107	-	0.044	4.93
2.418	-0.086	0.107	-0.195	-	-	0.105	-1.488	0.123	4.94
1.454	-	-	-	0.027	-0.105	-	-1.259	0.043	5.01
2.280	-0.078	-	-	0.014	-	-	-1.456	0.043	5.02
0.124	-	0.100	-0.173	-	-0.056	0.107	-	0.082	5.06
0.715	-0.070	-	-0.170	-	-0.043	0.087	-	0.079	5.20
0.559	-0.058	0.069	-	-	-	0.130	-	0.040	5.21
0.104	-	0.096	-0.186	0.017	-	0.112	-	0.078	5.26
0.101	-	-	-	0.044	-0.095	0.112	-	0.039	5.26
1.449	-	-	-0.168	0.014	-0.053	-	-1.236	0.078	5.30
0.707	-0.071	-	-0.182	0.011	-	0.091	-	0.077	5.33
0.727	-0.071	0.079	-0.195	-	-0.055	-	-	0.077	5.38
0.526	-0.055	-	-	0.039	-	0.119	-	0.034	5.52
0.062	-	0.067	-	0.045	-	0.134	-	0.034	5.56
0.765	-0.077	0.079	-0.214	-0.015	-	-	-	0.073	5.58
1.522	-	0.055	-	0.029	-	-	-1.362	0.033	5.59
2.299	-0.074	0.082	-	-	-	0.129	-1.505	0.073	5.59

2.193	-0.072	-	-	-	-0.087	0.104	-1.387	0.073	5.61
1.530	-	0.092	-	-	-0.100	0.123	-1.334	0.072	5.63
0.591	-0.057	0.054	-	-	-0.116	-	-	0.031	5.75
2.432	-0.087	0.091	-0.197	-	-0.045	-	-1.474	0.109	5.87
0.120	-	-	-0.156	0.031	-0.050	0.093	-	0.068	5.88
1.537	-	0.112	-0.172	-	-0.050	0.108	-1.324	0.108	5.90
2.320	-0.085	-	-0.170	-	-0.034	0.085	-1.385	0.108	5.91
2.564	-0.096	0.095	-0.216	-0.034	-	-	-1.531	0.108	5.93
0.748	-0.073	-	-0.183	-0.002	-0.051	-	-	0.067	5.94
0.583	-0.056	-	-	0.017	-0.109	-	-	0.026	5.99
2.360	-0.089	-	-0.181	-0.004	-	0.087	-1.406	0.106	6.00
1.537	-	0.109	-0.185	0.006	-	0.111	-1.339	0.105	6.08
0.130	-	0.081	-0.185	0.005	-0.065	-	-	0.064	6.14
2.192	-0.072	-	-	0.023	-	0.115	-1.416	0.064	6.15
0.109	-	0.053	-	0.025	-0.119	-	-	0.023	6.18
2.279	-0.073	0.065	-	-	-0.107	-	-1.460	0.062	6.27
1.424	-	-	-	0.036	-0.089	0.109	-1.238	0.062	6.28
1.511	-	0.080	-	0.033	-	0.134	-1.354	0.061	6.33
2.409	-0.091	-	-0.184	-0.016	-0.042	-	-1.414	0.097	6.61
2.232	-0.074	-	-	0.003	-0.100	-	-1.404	0.055	6.66
0.566	-0.059	0.040	-	0.022	-	-	-	0.014	6.73

0.555	-0.054	0.077	-	-	-0.102	0.120	-	0.053	6.83
0.687	-0.067	0.097	-0.183	-	-0.047	0.103	-	0.093	6.86
1.424	-	-	-0.155	0.023	-0.044	0.091	-1.221	0.091	6.98
