# Supplementary material for: Nest characteristics determine nest microclimate and affect breeding output in an Antarctic seabird, the Wilson’s storm-petrel
Source: PLoS One. 2019 Jun 13;14(6):e0217708. doi: 10.1371/journal.pone.0217708 (PMC6564424; doi:10.1371/journal.pone.0217708)
Supplement: S7 Table — Unscaled parameter estimates for each model are shown. Only models within 4 units of AICc are shown, due to the high number of possible models. Models used in model averaging are indicated in bold. (PDF) [file pone.0217708.s007.pdf]

S7 Table. Model selection for the effects of nest parameters on chick survival. Unscaled parameter estimates for each model are shown. Only models within 4 units of AICc are shown, due to the high number of possible models. Models used in model averaging are indicated in bold.

Intercept	log Entrance size	Cooling coefficient	Nest height	Northern entrance orientation	Eastern entrance orientation	Eastern nest site orientation	Nest depth	log TRI	WEI	Breeding season	R^2_p	ΔAICc
**2.075**	**-**	**-**	**-**	**-**	**1.425**	**-**	**-0.059**	**-**	**-**	**-**	**0.248**	**0.00**
**2.607**	**-**	**-**	**-**	**-**	**1.813**	**-1.220**	**-0.065**	**-**	**-**	**-**	**0.326**	**0.10**
**0.236**	**-**	**-**	**-**	**-**	**1.336**	**-**	**-**	**-**	**-**	**-**	**0.161**	**0.14**
**0.518**	**-**	**-**	**-**	**-**	**1.713**	**-1.052**	**-**	**-**	**-**	**-**	**0.235**	**0.40**
**5.417**	**-0.637**	**-**	**-**	**-**	**1.668**	**-**	**-**	**-**	**-**	**-**	**0.217**	**0.94**
**2.494**	**-**	**-**	**-**	**-0.702**	**1.744**	**-**	**-0.075**	**-**	**-**	**-**	**0.288**	**1.34**
**5.834**	**-0.652**	**-**	**-**	**-**	**2.084**	**-1.129**	**-**	**-**	**-**	**-**	**0.287**	**1.35**
**2.258**	**-**	**-**	**-**	**-**	**1.448**	**-**	**-0.064**	**-0.723**	**-**	**-**	**0.286**	**1.39**
**3.125**	**-**	**-**	**-**	**-**	**2.142**	**-1.417**	**-0.069**	**-**	**-**	**+**	**0.368**	**1.46**
**0.285**	**-**	**-**	**-**	**-**	**1.313**	**-**	**-**	**-0.558**	**-**	**-**	**0.191**	**1.70**
**0.500**	**-**	**-**	**-**	**-**	**1.483**	**-**	**-**	**-**	**-**	**+**	**0.190**	**1.71**
**2.369**	**-**	**-**	**-**	**-**	**1.571**	**-**	**-0.060**	**-**	**-**	**+**	**0.275**	**1.73**
**2.929**	**-**	**-**	**-**	**-**	**1.806**	**-1.247**	**-0.072**	**-0.704**	**-**	**-**	**0.359**	**1.77**
**0.857**	**-**	**-**	**-**	**-**	**1.955**	**-1.162**	**-**	**-**	**-**	**+**	**0.273**	**1.78**
1.041	-	-5.536	-	-	1.349	-	-	-	-	-	0.179	2.03
2.431	-	-	-	-	-	-	-0.053	-	-	-	0.088	2.13
0.780	-	-	-	-	-	-	-	-	-	-	0.000	2.15
0.204	-	-	-	-0.295	1.452	-	-	-	-	-	0.171	2.26
0.565	-	-	-	-	1.663	-1.015	-	-0.491	-	-	0.255	2.34

0.679	-	-	-0.040	-	1.286	-	-	-	-	-	0.166	2.39
4.788	-	-	-	-	1.432	-	-0.062	-	-2.460	-	0.253	2.41
2.378	-	-2.732	-	-	1.428	-	-0.056	-	-	-	0.252	2.44
3.411	-0.202	-	-	-	1.515	-	-0.049	-	-	-	0.251	2.46
2.221	-	-	-0.015	-	1.404	-	-0.059	-	-	-	0.249	2.54
0.411	-	-	-	-	1.337	-	-	-	-0.164	-	0.161	2.54
5.556	-0.648	-	-	-	1.639	-	-	-0.604	-	-	0.247	2.60
1.183	-	-	-0.054	-	1.624	-1.127	-	-	-	-	0.243	2.70
2.726	-	-	-	-0.279	1.861	-1.068	-0.070	-	-	-	0.331	2.70
5.059	-	-	-	-	1.833	-1.208	-0.067	-	-2.240	-	0.330	2.73
3.037	-	-	-0.038	-	1.754	-1.294	-0.063	-	-	-	0.330	2.73
1.051	-	-3.728	-	-	1.697	-1.004	-	-	-	-	0.242	2.75
2.777	-	-	-	-0.751	1.782	-	-0.083	-0.786	-	-	0.329	2.76
3.713	-0.166	-	-	-	1.893	-1.221	-0.057	-	-	-	0.328	2.77
2.584	-	0.226	-	-	1.815	-1.225	-0.065	-	-	-	0.326	2.84
5.388	-0.605	-	-	-	1.775	-	-	-	-	+	0.237	2.89
0.552	-	-	-	0.155	1.696	-1.144	-	-	-	-	0.237	2.91
5.905	-0.703	-	-	-0.446	1.883	-	-	-	-	-	0.235	2.94
0.416	-	-	-	-	1.712	-1.052	-	-	0.096	-	0.235	2.96
2.901	-	-	-	-0.796	1.962	-	-0.078	-	-	+	0.322	2.98
2.627	-	-	-	-	-	-	-0.057	-0.736	-	-	0.142	3.07
5.924	-0.612	-4.862	-	-	1.669	-	-	-	-	-	0.230	3.11
0.833	-	-	-	-	-	-	-	-0.621	-	-	0.047	3.21

5.678	-0.597	-	-	-	2.249	-1.208	-	-	-	+	0.314	3.23
3.499	-	-	-	-	2.122	-1.462	-0.076	-0.731	-	+	0.400	3.29
5.926	-0.640	-	-0.042	-	1.598	-	-	-	-	-	0.222	3.33
1.711	-	-7.774	-	-	1.558	-	-	-	-	+	0.222	3.33
2.548	-	-	-	-	1.582	-	-0.065	-0.714	-	+	0.311	3.33
6.134	-0.681	-	-	-	2.060	-1.121	-	-0.559	-	-	0.311	3.34
7.293	-0.658	-	-	-	1.682	-	-	-	-1.595	-	0.219	3.43
0.551	-	-	-	-	1.450	-	-	-0.554	-	+	0.218	3.45
1.615	-	-	-0.076	-	-	-	-	-	-	-	0.029	3.66
6.850	-0.672	-	-0.066	-	1.975	-1.255	-	-	-	-	0.299	3.73
1.075	-	-5.423	-	-	1.331	-	-	-0.556	-	-	0.207	3.77
2.993	-	-4.191	-	-0.753	1.757	-	-0.071	-	-	-	0.296	3.82
0.486	-	-	-	-0.362	1.639	-	-	-	-	+	0.204	3.87
0.915	-	-	-	-	1.902	-1.133	-	-0.501	-	+	0.293	3.90
0.964	-	-	-	-	-	-0.457	-	-	-	-	0.020	3.90
2.684	-	-	-	-	-	-0.534	-0.054	-	-	-	0.111	3.91
5.113	-	-	-	-0.697	1.754	-	-0.078	-	-2.373	-	0.292	3.93
1.536	-	-5.104	-	-	-	-	-	-	-	-	0.019	3.93
6.079	-0.636	-2.671	-	-	2.063	-1.083	-	-	-	-	0.291	3.98
0.246	-	-	-	-0.292	1.426	-	-	-0.555	-	-	0.200	3.99
