# Supplementary material for: Nest characteristics determine nest microclimate and affect breeding output in an Antarctic seabird, the Wilson’s storm-petrel
Source: PLoS One. 2019 Jun 13;14(6):e0217708. doi: 10.1371/journal.pone.0217708 (PMC6564424; doi:10.1371/journal.pone.0217708)
Supplement: S8 Table — Chick growth rate was measured as the % body mass change. Scaled parameter estimates for each model are shown. Models used in model averaging are indicated in bold. (PDF) [file pone.0217708.s008.pdf]

S8 Table. Model selection for the effects of weather conditions and breeding season on chick growth rate. Chick growth rate was measured as the % body mass change. Scaled parameter estimates for each model are shown. Models used in model averaging are indicated in bold.

Intercept	Snow cover	Northern wind direction	Eastern wind direction	Wind speed	Breeding season	Northern wind direction x Breeding season	Eastern wind direction x Breeding season	Wind speed x Breeding season	R^2_p	ΔAICc
**0.068**	**0.094**	**-**	**-**	**-0.036**	**+**	**-**	**-**	**+**	**-0.013**	**0.00**
**0.074**	**-**	**-**	**-0.003**	**-0.030**	**+**	**-**	**+**	**+**	**-0.014**	**1.21**
**0.072**	**-**	**-**	**-**	**-0.031**	**+**	**-**	**-**	**+**	**-0.009**	**1.39**
0.067	0.087	-	0.002	-0.037	+	-	-	+	-0.013	2.08
0.067	0.096	0.001	-	-0.037	+	-	-	+	-0.013	2.12
0.065	-	-	0.009	-0.036	+	-	-	+	-0.010	2.27
0.073	0.050	-	-0.004	-0.032	+	-	+	+	-0.014	2.79
0.062	0.103	0.008	-	-0.041	+	+	-	+	-0.014	3.08
0.079	-	-0.005	-0.006	-0.026	+	-	+	+	-0.014	3.13
0.072	-	0.000	-	-0.031	+	-	-	+	-0.009	3.52
0.069	-	0.004	-0.001	-0.034	+	+	+	+	-0.015	3.85
0.065	0.088	0.002	0.003	-0.038	+	-	-	+	-0.013	4.20
0.064	-	0.002	0.009	-0.038	+	-	-	+	-0.011	4.37
0.092	-	-	-	-	-	-	-	-	0.000	4.42
0.054	-	0.011	0.014	-0.046	+	+	-	+	-0.012	4.74
0.056	0.084	0.011	0.007	-0.046	+	+	-	+	-0.014	4.76

0.076	0.045	-0.003	-0.006	-0.029	+	-	+	+	-0.014	4.87
0.087	0.058	-	-	-	-	-	-	-	-0.002	4.88
0.068	-	0.005	-	-0.034	+	+	-	+	-0.010	4.97
0.067	0.044	0.006	-0.001	-0.036	+	+	+	+	-0.016	5.63
0.092	-	-0.006	-	-	-	-	-	-	-0.001	5.87
0.089	-	-	-	-	+	-	-	-	0.000	6.37
0.092	-	-	-0.002	-	-	-	-	-	0.000	6.42
0.092	-	-	-	-0.001	-	-	-	-	0.000	6.47
0.087	0.066	-	-0.004	-	-	-	-	-	-0.002	6.60
0.087	0.053	-0.004	-	-	-	-	-	-	-0.002	6.67
0.087	0.065	-	-	-0.004	-	-	-	-	-0.002	6.70
0.094	-	-	-0.013	-	+	-	+	-	-0.004	6.90
0.088	0.063	-	-	-	+	-	-	-	-0.002	6.92
0.099	-	-0.009	-0.015	-	+	-	+	-	-0.005	7.80
0.092	-	-0.005	-0.001	-	-	-	-	-	-0.001	7.91
0.092	-	-0.006	-	-0.001	-	-	-	-	-0.001	7.94
0.091	-	-0.005	-	-	+	-	-	-	-0.001	7.95
0.086	0.077	-	-0.006	-0.005	-	-	-	-	-0.002	8.24
0.091	0.089	-	-0.008	-	+	-	-	-	-0.002	8.25
0.087	-	-	-	-0.003	+	-	-	-	0.000	8.32
0.094	0.050	-	-0.014	-	+	-	+	-	-0.004	8.42

0.090	-	-	-0.001	-	+	-	-	-	0.000	8.44
0.092	-	-	-0.002	-0.001	-	-	-	-	0.000	8.48
0.087	0.060	-0.003	-0.004	-	-	-	-	-	-0.002	8.49
0.087	0.060	-0.004	-	-0.003	-	-	-	-	-0.002	8.57
0.090	0.061	-0.005	-	-	+	-	-	-	-0.002	8.59
0.086	0.065	-	-	-0.004	+	-	-	-	-0.002	8.81
0.097	-	-	-0.014	0.003	+	-	+	-	-0.004	8.91
0.107	-	-0.012	-0.019	0.009	+	-	+	-	-0.006	9.07
0.098	0.044	-0.008	-0.016	-	+	-	+	-	-0.006	9.48
0.094	0.089	-0.006	-0.008	-	+	-	-	-	-0.003	9.85
0.099	-	-0.009	-0.015	-	+	+	+	-	-0.005	9.93
0.092	-	-0.005	-0.002	-0.001	-	-	-	-	-0.001	10.01
0.092	-	-0.006	-0.002	-	+	-	-	-	-0.001	10.02
0.090	-	-0.005	-	-0.001	+	-	-	-	-0.001	10.04
0.092	-	-0.006	-	-	+	+	-	-	-0.001	10.04
0.089	0.090	-	-0.008	-0.004	+	-	-	-	-0.003	10.19
0.086	0.072	-0.003	-0.005	-0.005	-	-	-	-	-0.003	10.23
0.088	-	-	-0.001	-0.003	+	-	-	-	0.000	10.41
0.095	0.048	-	-0.015	0.002	+	-	+	-	-0.004	10.54
0.089	0.063	-0.004	-	-0.002	+	-	-	-	-0.002	10.65
0.091	0.061	-0.005	-	-	+	+	-	-	-0.002	10.72

0.105	0.028	-0.011	-0.019	0.008	+	-	+	-	-0.006	11.07
0.107	-	-0.013	-0.019	0.009	+	+	+	-	-0.006	11.21
0.099	0.045	-0.009	-0.016	-	+	+	+	-	-0.006	11.63
0.094	0.089	-0.007	-0.009	-	+	+	-	-	-0.003	11.94
0.092	0.089	-0.005	-0.008	-0.002	+	-	-	-	-0.003	11.96
0.092	-	-0.007	-0.002	-	+	+	-	-	-0.001	12.12
0.091	-	-0.005	-0.001	-0.001	+	-	-	-	-0.001	12.13
0.091	-	-0.006	-	-0.001	+	+	-	-	-0.001	12.15
0.089	0.062	-0.005	-	-0.002	+	+	-	-	-0.002	12.80
0.106	0.029	-0.012	-0.019	0.008	+	+	+	-	-0.006	13.23
0.093	0.090	-0.006	-0.009	-0.002	+	+	-	-	-0.003	14.08
0.092	-	-0.006	-0.002	-0.001	+	+	-	-	-0.001	14.25
