# Supplementary material for: Nest characteristics determine nest microclimate and affect breeding output in an Antarctic seabird, the Wilson’s storm-petrel
Source: PLoS One. 2019 Jun 13;14(6):e0217708. doi: 10.1371/journal.pone.0217708 (PMC6564424; doi:10.1371/journal.pone.0217708)
Supplement: S1 File — The quantification of the nest insulation and a sensitivity analysis of different measurement durations. (PDF) [file pone.0217708.s016.pdf]

S1 File. Additional information on nest insulation

Quantification of the nest insulation

To quantify nest insulation, we calculated the mean cooling coefficient of each nest, by fitting Newton's law of cooling through each set of logged temperature values obtained from a single experiment in one nest, according to the equation:

$$T(t) = T_c + (T(0) - T_c) \cdot e^{-kt} \qquad (1)$$

where $T(t)$ is the nest temperature at time t, T_c is the horizontal asymptote, i.e. the constant nest floor temperature, $T(0)$ is the nest floor temperature at the first measurement, at t = 0 and k is the cooling coefficient. From the fitted models, we derived the cooling coefficient, k, as a quantification of insulation, with a lower cooling coefficient meaning a better insulation. As we repeated each experiment four times per nest, we calculated the average cooling coefficient of each nest.

Sensitivity analysis of different measurement durations

The 20 min duration was *a priori* established as the minimum duration to fit a cooling curve based on Newton's law of cooling and to obtain estimations of the cooling coefficient (equation 1 analyses section). However, the logger was occasionally left in the nest for up to 60 min due to logistic circumstances in the field. We compared models fitted to a data set with 30, 25 or a subset of 20 minutes (Fig 1), and found that a shorter measurement duration would generally lead to a slight overestimation of the horizontal asymptote (i.e. the floor temperature before warming or after cooling down) and the cooling coefficient. However, this variation was well within the variation observed between different repetitions of the experiment (Table 1). Hence, we decided *posteriori* that 20 minutes would be the considered the minimum duration of the experiment.

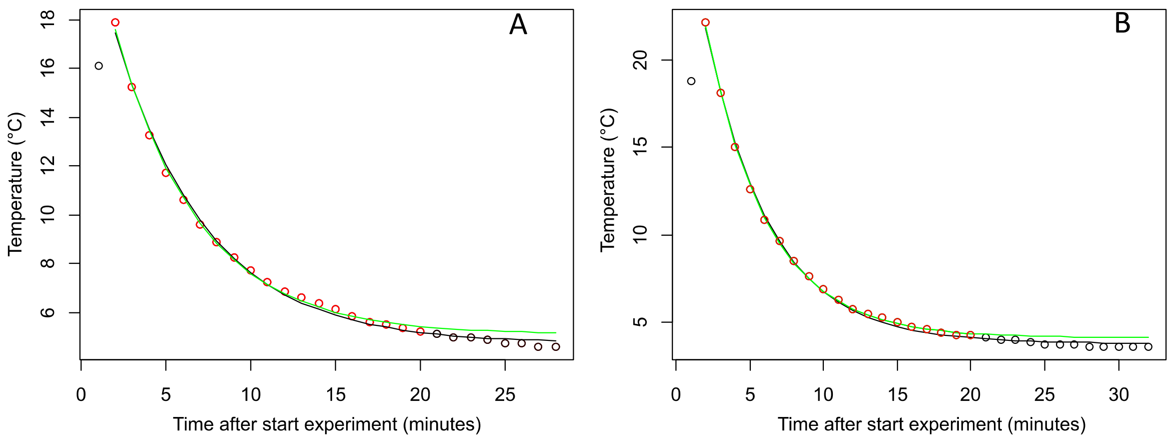

S2 Fig 1. Newton's law of cooling. Two cooling curves obtained during experiments in the same nest, but on different days. Circles represent recorded temperature values. Red circles are values used for fitting a model based on a subset of 20 minutes of the data. Lines represent fitted model based on all (black) or only 20 minutes (green) of the data. Derived parameter estimates are provided in S2 Table.

S2 Table 1. Parameters estimates from fitting Newton's law of cooling. A cooling curve was fitted to either the full set (28 or 32 minutes) or a subset (20 minutes) of the data obtained from two experiments performed in the same nest on different days.

Figure	1A		1B	
Duration (minutes)	20	28	20	32
Horizontal asymptote (°C)	4.12	3.78	5.11	4.74
Cooling coefficient	0.238	0.224	0.202	0.185
