# Supplementary material for: Nest characteristics determine nest microclimate and affect breeding output in an Antarctic seabird, the Wilson’s storm-petrel
Source: PLoS One. 2019 Jun 13;14(6):e0217708. doi: 10.1371/journal.pone.0217708 (PMC6564424; doi:10.1371/journal.pone.0217708)
Supplement: S2 File — The calculations of chick growth, the effect of nest air temperature on chick growth in 2018 and the effect of nest characteristics on chick growth. (PDF) [file pone.0217708.s017.pdf]

S2 File. Additional analyses of chick growth

The calculations of chick growth

For each time interval between the two consecutive measurements, we calculated chick growth rate as the daily percentage body mass increase, according to the equation:

$$\%\, body\ mass\ increase = \frac{M_n - M_{n-1}}{M_{n-1} \cdot \Delta days} \cdot 100 \qquad (1)$$

where M_n is the body mass (g) at a specific nest check (n) and $\Delta days$ is the number of days between the two consecutive nest checks (n and $n-1$). We choose to use daily percentage body mass increase between two consecutive nest checks, instead of the more commonly used sigmoid growth curve. We selected this approach, since the daily percentage body mass increase accurately represents the stochasticity in growth, due to for example weather conditions, whereas a smoothed growth curve could mask these effects (but see below for additional analyses of chick growth). We only included chicks older than 5 days in the analyses, since chicks are capable of thermoregulation after 5 days after hatching [52,63] and hence a trade-off between energy invested in either thermoregulation or growth was expected to be only apparent in older chicks. In addition, we only included chicks younger than 18 days, as chicks reach their peak body mass between 18 and 46 days, followed by natural loss of mass independent of environmental conditions [63]. Hence, to ensure chicks involved in the analyses had not reached their peak mass already, we set the maximum age to 18 days. For the chicks which we found after hatching (and thus did not know the exact hatching date), we derived their age using the relationship between wing length and age, obtained by modelling wing length with the age of 20 chicks with a known hatching date (accuracy ± 3 days, due to nest check frequency), according to the equation:

$$Age = \frac{a}{1+e^{-\left(b+cL_{wing}\right)}} \qquad (2)$$

Where Age is the estimated age of the chick in days, L_{wing} is the wing length in mm, a is the horizontal asymptote, i.e. the final wing length, b is the second parameter and c is the growth parameter. The resulting model parameters were $a =$162.4 ± 3.10, $b =$ -3.18 ± 0.07 and $c =$ 0.10 ± 0.003 (N = 221).

The effect of nest air temperature on chick growth in 2018

To examine the effect of nest air temperature on chick growth in 2018, we modelled chick growth as the percentage body mass increase between measurements with the mean nest air temperature of the previous seven days, using a linear mixed effect model with nest ID as the random

effect to correct for repeated sampling of individuals over time. We tested the significance of the parameter by bootstrapping the model fitting.

The effect of nest air temperature on growth rate was -0.174 ± 0.114, and could be considered as a trend ($p = 0.055$, $N_{nest} = 17$, $N_{per\ nest} = 1\text{-}6$, S9 Figure 1).

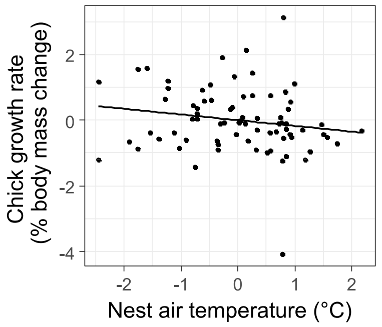

S9 Fig 1. Chick growth in response to nest temperature. The effect of nest air temperature on chick growth in 2018.

Even though this effect is only a trend, the negative effect suggests that chick growth is favoured when nest air temperatures are low. This does not support our hypothesis that chick growth is favoured by high nest air temperatures. Most likely, nest air temperature did not critically affect chick growth in this study.

The effect of nest characteristics on chick growth

To examine the effect of nest characteristics on chick growth, we obtained the chick growth parameters for each nest, by fitting a non-linear model described by the function:

$$M_{body} = \frac{a}{1+e^{-(b+c\cdot Age)}} \quad (1)$$

In this function, M_{body} is the estimated body mass of the chick in g, Age is the age of the chick in days from hatching, a is the horizontal asymptote, i.e. the final body mass, b is the second parameter describing the curve and c is the growth parameter. We started modelling the growth parameter with the nest parameters that were associated with the nest specific thermal microclimate and snow blocking, i.e. TRI, WEI, eastern nest site orientation, northern and eastern entrance orientation, entrance size, cooling coefficient, nest height and nest depth, and breeding season to account for inter-annual differences. To correct for skewness of the data, we log-transformed nest entrance size, nest width and the TRI. We performed model averaging on the best models to obtain weighted parameter estimates (S9 Table 1) [77]. We tested the significance of the parameters by bootstrapping the model fitting of a model including all averaged parameters (S9 Table 1 and S9 Table 2).

The best models predicting chick growth included the parameters: nest height, breeding season, cooling coefficient and TRI (S9 Table 1). Chick growth was significantly higher in 2018 and was favoured in higher nests (S9 Table 2 and S9 Figure 2).

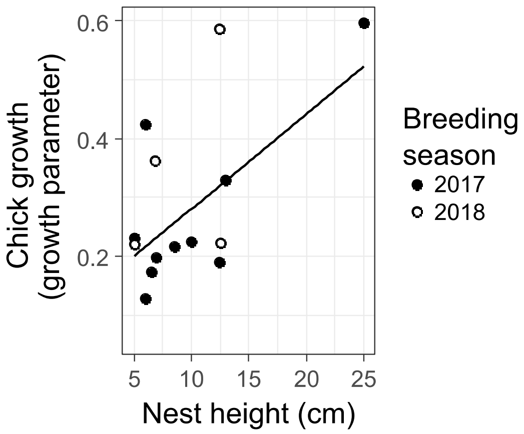

S9 Fig 2. Chick growth in response to nest height. The effect of nest height on chick growth in 2017 (filled circles) and 2018 (circles). Estimates, p-values and the relative importance of each nest parameter are provided in S9 Table 2.

Since nest height significantly reduced the quality of the thermal nest microclimate, this result might indicate that chicks are favoured by low nest air temperatures. This does not support our hypothesis that chick growth is favoured by high nest air temperatures. Hence, most likely, nest air temperature does not critically affect chick growth.

S9 Table 1. Model selection for the effects of nest characteristics and breeding season on chick growth. Chick growth rate was measured as the growth parameter. Unscaled parameter estimates for each model are shown. Only models within 4 units of AICc are shown, due to the high number of possible models. Models used in model averaging are indicated in bold.

**Intercept**	**log Entrance size**	**Cooling coefficient**	**Nest height**	**Northern entrance orientation**	**Eastern entrance orientation**	**Eastern nest site orientation**	**Nest depth**	**log Terrain Ruggedness Index**	**Wind Exposition Index**	**log Nest width**	**Breeding season**	**R^2_p**	**ΔAICc**
**0.118**	**-**	**-**	**0.016**	**-**	**-**	**-**	**-**	**-**	**-**	**-**	**-**	**-0.178**	**0.00**
**-379.3**	**-**	**1.243**	**0.019**	**-**	**-**	**-**	**-**	**-**	**-**	**-**	**0.188**	**-0.354**	**0.93**
**-224.3**	**-**	**-**	**0.017**	**-**	**-**	**-**	**-**	**-**	**-**	**-**	**0.111**	**-0.245**	**1.38**
**0.007**	**-**	**-**	**0.026**	**-**	**-**	**-**	**-**	**-0.144**	**-**	**-**	**-**	**-0.242**	**1.48**
**0.277**	**-**	**-**	**-**	**-**	**-**	**-**	**-**	**-**	**-**	**-**	**-**	**0.000**	**1.88**
0.035	-	0.558	0.017	-	-	-	-	-	-	-	-	-0.207	2.83
0.063	-	-	0.020	-	0.057	-	-	-	-	-	-	-0.198	3.13
0.049	-	-	0.015	-	-	-	0.002	-	-	-	-	-0.191	3.37
-335.7	-	1.351	0.028	-	-	-	-	-0.138	-	-	0.166	-0.408	3.39
0.285	-	-	0.015	-	-	-	-	-	-	-0.057	-	-0.188	3.50
0.124	-	-	0.016	0.016	-	-	-	-	-	-	-	-0.182	3.68
0.111	-	-	0.017	-	-	0.012	-	-	-	-	-	-0.180	3.75
0.175	-0.006	-	0.016	-	-	-	-	-	-	-	-	-0.180	3.78
0.006	-	-	0.016	-	-	-	-	-	0.106	-	-	-0.179	3.79
-193.4	-	-	-	-	-	-	-	-	-	-	0.096	-0.050	3.81

-0.136	-	0.796	0.029	-	-	-	-	-0.175	-	-	-	-0.297	3.83
0.609	-	-	-	-	-	-	-	-	-	-0.120	-	-0.045	3.93

S9 Table 2. Effects of nest characteristics on the chick growth parameter, N_{nest} = 15

Parameter	Estimate	±	SE	Relative importance	p-value
Intercept	-116.9		190.0		0.030
**Nest height**	**0.016**		**0.010**	**0.87**	**0.020**
**Breeding season**	**0.058**		**0.094**	**0.38**	**0.032**
Cooling coefficient	0.261		0.589	0.21	0.117
log Terrain Ruggedness Index	-0.023		0.069	0.16	0.139

Weighted averages of the parameter estimates were calculated using all models within 2 AICc units of the model with the lowest AICc value (Table S7-1). The parameter estimates were calculated using the full-model averaging method [82]. Parameters are ordered according to their relative importance, i.e. the sum of the Akaike weights of all the models with ΔAICc < 2 containing this parameter [77]. Significant effects ($p < 0.05$) are indicated in bold.

65
